# Serelaxin alleviates cardiac fibrosis through inhibiting endothelial-to-mesenchymal transition via RXFP1

**DOI:** 10.7150/thno.38640

**Published:** 2020-03-04

**Authors:** Tim Wilhelmi, Xingbo Xu, Xiaoying Tan, Melanie S. Hulshoff, Sabine Maamari, Samuel Sossalla, Michael Zeisberg, Elisabeth M. Zeisberg

**Affiliations:** 1 Department of Cardiology and Pneumology, University Medical Center of Göttingen, Georg-August University, Göttingen, Germany.; 2 Department of Nephrology and Rheumatology, University Medical Center of Göttingen, Georg-August University, Göttingen, Germany.; 3 DZHK (German Centre for Cardiovascular Research, partner site Göttingen, Germany).; 4 Laboratory for Cardiovascular Regenerative Medicine, Department of Pathology and Medical Biology, University of Groningen, Groningen, The Netherlands.; 5 Department of Internal Medicine II, University Medical Center Regensburg, Regensburg, Germany.

**Keywords:** Serelaxin, EndMT, fibrosis, Notch, histone methylation

## Abstract

**Rationale**: Cardiac fibrosis is an integral constituent of every form of chronic heart disease, and persistence of fibrosis reduces tissue compliance and accelerates the progression to heart failure. Relaxin-2 is a human hormone, which has various physiological functions such as mediating renal vasodilation in pregnancy. Its recombinant form Serelaxin has recently been tested in clinical trials as a therapy for acute heart failure but did not meet its primary endpoints. The aim of this study is to examine whether Serelaxin has an anti-fibrotic effect in the heart and therefore could be beneficial in chronic heart failure.

**Methods**: We utilized two different cardiac fibrosis mouse models (ascending aortic constriction (AAC) and Angiotensin II (ATII) administration via osmotic minipumps) to assess the anti-fibrotic potential of Serelaxin. Histological analysis, immunofluorescence staining and molecular analysis were performed to assess the fibrosis level and indicate endothelial cells which are undergoing EndMT. *In vitro* TGFβ1-induced endothelial-to-mesenchymal transition (EndMT) assays were performed in human coronary artery endothelial cells and mouse cardiac endothelial cells (MCECs) and were examined using molecular methods. Chromatin immunoprecipitation-qPCR assay was utilized to identify the Serelaxin effect on chromatin remodeling in the *Rxfp1* promoter region in MCECs.

**Results**: Our results demonstrate a significant and dose-dependent anti-fibrotic effect of Serelaxin in the heart in both models. We further show that Serelaxin mediates this effect, at least in part, through inhibition of EndMT through the endothelial Relaxin family peptide receptor 1 (RXFP1). We further demonstrate that Serelaxin administration is able to increase its own receptor expression (RXFP1) through epigenetic regulation in form of histone modifications by attenuating TGFβ-pSMAD2/3 signaling in endothelial cells.

**Conclusions**: This study is the first to identify that Serelaxin increases the expression of its own receptor RXFP1 and that this mediates the inhibition of EndMT and cardiac fibrosis, suggesting that Serelaxin may have a beneficial effect as anti-fibrotic therapy in chronic heart failure.

## Introduction

Cardiac fibrosis is an integral constituent of every form of chronic heart disease and leads to an increased wall stiffness and diastolic dysfunction. Regardless of the pathogenesis of cardiac fibrosis, activated fibroblasts mediate fibrogenesis by excessive extracellular matrix production 1-3. However, currently there is no specific therapy available in the clinic yet to block the progression of cardiac fibrosis, albeit several groups have shown different anti-fibrotic strategies in proof-of-concept studies 4-8.

Serelaxin is a recombinant form of the human hormone Relaxin-2, which is a 6kDa polypeptide hormone known to have various physiological functions such as mediating renal vasodilation during pregnancy. Furthermore, it is able to soften the cervix, increase both the flexibility of the interpubic ligament and motility of male sperm and is, most importantly, involved in organ remodeling (of the skin, lung, liver, kidney and heart) by inducing degradation of extracellular matrix 9-13. Relaxin peptides bind to one of their cognate receptors called Relaxin family peptide receptors (RXFPs) 1 - 4. RXFPs are expressed in heart tissue, blood vessels, and the kidneys 12-14 and trigger various pathways resulting in an induction of multiple functional products such as vascular endothelial growth factor (VEGF), endothelial nitric oxide synthase (eNOS) and matrix metalloproteinases (MMPs) 13,15. In Relaxin-knockout mice, an increased deposition of collagen I was observed in the left ventricle 16. In another study, an anti-fibrotic effect of Relaxin could be confirmed; however the molecular mechanisms have not yet been addressed 12,17,18. Nevertheless, with its vasodilating, anti-inflammatory and anti-fibrotic effects; Relaxin has gained attention in cardiovascular research as this single compound targets different aspects of heart failure.

In an international, double-blinded and placebo-controlled study, the effect of Serelaxin was examined in 1161 hospitalized patients with acute heart failure. Patients received an intravenous infusion of Serelaxin (30 μg/kg per day) or placebo infusion for 48 hours. In this study, the administration of Serelaxin showed a significant improvement of dyspnea (shortness of breath) and a 37% reduction in both cardiovascular and overall mortality within 6 months as compared to the placebo control group. Furthermore, worsening of heart failure events could be reduced by 47% after day 5 19,20. Cardiac output was not significantly changed by Serelaxin 21. An additional phase III trial (RELAX-AHF 2) including 6,600 patients hospitalized for acute heart failure was designed to confirm whether Serelaxin has significant effects on reducing cardiovascular death and worsening heart failure episodes. However, it failed to reduce the previous reported cardiovascular death after 6 months or worsening of heart failure after 5 days 22. Here, we explored the effect of Serelaxin on cardiac fibrosis. In this respect, an anti-fibrotic effect of Relaxin has been observed in both kidney and heart using an experimental model of spontaneously hypertensive rats 23. However, no pathomechanistic insights were provided by this study. *In vitro* studies demonstrated an anti-transforming growth factor β (TGFβ)/Smad3-mediated protective effect of Relaxin on fibroblast activation 24. Because the pro-fibrotic mechanism of endothelial-to-mesenchymal transition (EndMT) is also Smad3-mediated, these studies further suggest an inhibitory and anti-fibrotic effect of Serelaxin with respect to EndMT.

Besides the TGFβ pathway, several other molecular signaling pathways such as Notch, Wnt, Erk1/2, p38, NF-κB, etc. can also independently or synergistically regulate EndMT 25. Notch signaling is involved in developmental EndMT during embryonic formation of the heart 26,27 but also in pathogenic EndMT during tumor development and fibrogenesis 28,29. In this aspect, Notch has been reported to preserve endothelial cell properties and attenuate EndMT, and that this can be affected by Relaxin 30,31. The Notch signaling pathway is activated via its ligand Jagged1 binding to the extracellular domain of the Notch1 receptor. This leads to cleavage of the Notch intracellular domain (NICD) by a γ-secretase and to translocation into the nucleus where downstream target genes are activated.

EndMT is a cellular transformation process by which endothelial cells lose endothelial and gain mesenchymal cell characteristics (e.g. loss of CD31 expression and gain of α-SMA). While this mechanism is a physiological cell transformation process during embryonic heart development (which allows the formation of the endocardial cushion and the heart valves from endocardial cells of the atrioventricular canal), EndMT has recently gained attention because of its contribution to cardiac fibrosis and fibrogenesis of other organs such as the lung, kidney and liver 7,32-38.

Here we aimed to explore the anti-fibrotic potential of Serelaxin and its function in inhibiting EndMT both *in vitro* and *in vivo*.

## Material and Methods

### Animal welfare and ethics statement

All animal experiments complied with ethical regulations and were conducted according to the animal experimental protocols, which were approved by the Institutional Review Board of the University of Göttingen and the responsible government authority of Lower Saxony (Germany). All animal procedures conformed to the guidelines from Directive 2010/63/EU of the European Parliament on the protection of animals used for scientific purposes. Animal surgery experimental protocols are described in the following texts.

### Ascending aortic constriction

Ascending aortic constriction (AAC) was performed under anesthesia in 12-week-old C57BL/6N mice (each group was composed of half male and half female mice), weighing between 25-30 g with a mixture of medetomidine (0.5 mg/kg), midazolam (5 mg/kg) and fentanyl (10 mg/kg) at 0.1 ml NaCl intraperitoneally before the surgery. In these mice, constriction of the ascending aorta was performed with a 24-gauge needle as previously described 38. Sham operation was performed under the same conditions except for the aortic banding. Echocardiography of the aortic pressure gradient was performed to verify aortic constriction, and all mice were sacrificed four weeks after operation by cervical dislocation under anesthesia.

### Echocardiographic measurement of the pressure gradient

Pressure gradients were measured according to the previously described protocol 39 using a Vevo 2100 (FUJIFILM VisualSonics, Toronto, Canada) system with a 30-MHz respiration- and ECG-controlled probe under anesthesia with 1.5% isoflurane by inhalation during the whole procedure. The ascending aorta was visualized with 2-D and color flow imaging. The distal ascending aortic flow velocity (distal to constriction in AAC mice) was measured by pulsed wave (PW) Doppler to assess the pressure gradient, which was estimated using the modified Bernoulli equation (pressure gradient = 4 × velocity^2^). The investigator was blinded with respect to treatment.

### Application of Serelaxin and PBS via osmotic minipump implantation

Serelaxin was applied at three different dosages (50, 200 and 500 µg/kg bodyweight/day) for 28 days. PBS was used as a vehicle control. Both Serelaxin and PBS were administered to animals using an osmotic minipump (Model 1004 ALZET, Cupertino, CA, USA). The minipumps were implanted during AAC operation by cutting the skin median of the dorsal neck. Skin was mobilized carefully from the muscle fascia. Afterwards the minipump was put into the skin pocket and the wound was closed by three sutures.

### Application of Angiotensin II via osmotic minipump implantation

14- to 16-week-old C57BL/6N mice between 25-30 g were used for Angiotensin II (ATII) experiments. ATII (1.5 mg/kg per day) plus Serelaxin (500 µg/kg bodyweight) or PBS as vehicle control were administered to animals at the same time using an osmotic minipump (ALZET Model 1002 for 2 weeks and 1004 for 4 weeks). The mice were anesthetized with 3% to 4% of isoflurane by inhalation during the whole procedure.

### Histological assessment of cardiac fibrosis

The percent area of left ventricular myocardial fibrosis was quantified using cellSens software. Perivascular fibrosis was defined as fibrotic lesions surrounding vessels of >100 µm.

### Immunofluorescence staining

Paraffin-embedded heart tissues were sectioned at 3 µm thickness, deparaffinized and rehydrated by xylol and graded alcohol series prior to staining. Antigen retrieval was performed with citrate buffer (Dako Retrieval Solution (10x) pH6, Agilent Dako, Santa Clara, CA, USA) for 40 minutes and thereafter washed with PBS for 10 minutes. The slides were blocked in 1% BSA in PBS at room temperature for 30 minutes before incubating with a primary antibody at 4°C overnight. The fluorescence labeled secondary antibodies were added and incubated at room temperature for 45 minutes. The cell nucleus was visualized with DAPI (1 mg/ml, #6335.1, Carl Roth, Karlsruhe, Germany) with 1:1000 dilution in PBS at room temperature for 5 minutes. Microscopic pictures were acquired using an Olympus Confocal Microscope FV-1000 and Fluoview program. The acquired pictures were processed using Photoshop CS6 software. The primary and secondary antibodies used in this study with dilution factors are listed below:

### Chromatin immunoprecipitation (ChIP)-qPCR

Chromatin immunoprecipitation for histone modifications was performed as previously described 40. Briefly, the chromatin was cross-linked with paraformaldehyde for 10 minutes and immunoprecipitation was performed with antibody dilutions listed below. The pull-down DNA was dissolved with 100 µl of elution buffer and 2 µl of DNA sample was used for each qPCR reaction with the real-time PCR primers (sequence listed below).

### Cell culture experiments and transfections

Human coronary artery endothelial cells (HCAECs, PromoCell, Heidelberg, Germany) and mouse cardiac endothelial cells (MCECs, CellLutions, Burlington, Ontario, Canada) were kept at 37°C supplemented with 5% CO₂. Every other day the cell medium was changed and supplemented with 10 ng/ml TGFβ1 (R&D Systems, Minneapolis, USA) or 20- 200 ng/ml Serelaxin (Novartis, Switzerland). For Notch inhibition experiment, DAPT (Sigma-Aldrich, St. Louis, CA, USA) was supplemented into the cell culture medium (20 µg/ml) and replaced every second day. A total cell number of 2 × 10^5^ was seeded onto 6-well-plates, incubated for 24 hours and starved with basal medium before treatment. Cell samples were collected at 2 or 4 days after treatment.

For transfection experiments, HCAECs and MCECs were seeded onto 10 cm plates (Griner), cultured overnight and transfected with Lipofectamine 2000 (Life Technologies, Carlsbad, CA, USA) according to the manufacturer's instructions 41. Briefly, 2.5 μg of shRNA-plasmid or 40 pmol of siRNA (Origene, Rockville, MD, USA) and Lipofectamine 2000 were mixed in a ratio of 1:2 in a total volume of 500 μl of Opti-MEM (Life Technologies), and then the transfection complex was formed by incubating 20 minutes at room temperature. Then, the DNA-Lipofectamine complex was added to the cells in endothelial cell basic medium. After 3 hours of incubation, the medium was replaced by growth medium. TGFβ1 treatment started one day after transfection, and cells were collected for gene expression analysis after 4 days of TGFβ1 treatment.

### RNA extraction and quantitative real-time PCR

Tissue was first shredded using TissueLyser LT (Qiagen, Hilden, Germany). Total RNA was extracted from cells or shredded tissue by PureLink RNA kit (Life Technologies, Carlsbad, USA) following the manufacturer's protocol. Prior to cDNA synthesis using the SuperScript II system (Life Technologies, Carlsbad, USA), 1 µg of total RNA was digested with DNAse I (Sigma Aldrich, St. Louis, CA, USA). qRT-PCR was performed with SYBR Master Mix (Life Technologies, Carlsbad, USA) in 20µl of final reaction volume, and 2 µl of diluted cDNA (1:20) was used as a template and run on a StepOne Plus real-time PCR system (Life Technologies) with real-time PCR primers (sequences listed below). Measurements were standardized to the GAPDH reaction using delta delta Ct methods.

### qPCR array

A human EMT Signaling Pathway PCR array (PAHS-090Z, SABiosciences, Frederick, MD, USA) was used to analyze the expression changes of genes related to EndMT in HCAECs. Briefly, 5 µg of total RNA was used for first-strand cDNA synthesis with the RT^2^ first strand kit (Qiagen). The PCR array was carried out following the manufacturer's instructions using the ready-to-use RT^2^-qPCR master mix (RT^2^-SYBR® Green/Fluorescein qPCR master mix, SABiosciences). 20µl master mix was added into each well containing pre-dispensed, gene-specific primer pairs and run on a StepOne Plus realtime PCR system (Life technologies). Data analysis was performed using the web-based standard RT PCR array suite (SABiosciences).

### Protein extraction and Western blotting

Cells and tissue were lysed with NP40 lysis buffer (Life Technologies) containing protease inhibition cocktail (Roche, Mannheim, Germany). Tissue was shredded by Tissue Lyser LT (Qiagen). Protein samples were loaded on a 4-12% SDS/PAGE gel and transferred onto a nitrocellulose membrane (GE Healthcare, Buckinghamshire, UK). After blocking with 5% dry milk in TBST solution (TBS pH 7.2, 0.1% Tween-20), the membrane was incubated with primary antibody solution (diluted in 2 % dry milk in TBST, dilution factors are indicated in the following table) at 4°C overnight. Next day, after three time washing with 2% dry milk in TBST, the membrane was incubated with secondary antibody (horseradish peroxidase-conjugated antibodies (Cell Signaling, Danvers, MA, USA)) diluted in 2% dry milk in TBST for 1 hour at room temperature and finally washed three times. Signals were detected using a chemiluminescent kit (Santa Cruz Biotechnology, Santa Cruz, CA, USA).

### Human tissue

All procedures were performed conform to the World Medical Association Declaration of Helsinki and the permission of human tissue usage was approved by the local ethics committee. Left ventricular myocardial tissue was obtained from end-stage heart failure patients at the time of heart transplantation or from donors without heart disease in cases when the heart could not be transplanted for technical reasons.

### Statistical analysis

Means were calculated and plotted along with standard error bars. All statistical analyses were done using GraphPad Prism software version 6.0 (GraphPad). All data were first analyzed by Student t-test for single comparison and one-way ANOVA with Bonferroni post-hoc analysis was used for comparing multiple groups. Significant differences between means were considered statistically significant when the p-value is smaller or equal to 0.05. Three or more biological replicates were included for all experiments. Overall survival was analyzed by using a Kaplan-Meier survival method with a log rank test to determine statistical differences.

## Results

### Serelaxin ameliorates cardiac fibrosis in two independent mouse models of pressure overload

**Figure 1:**
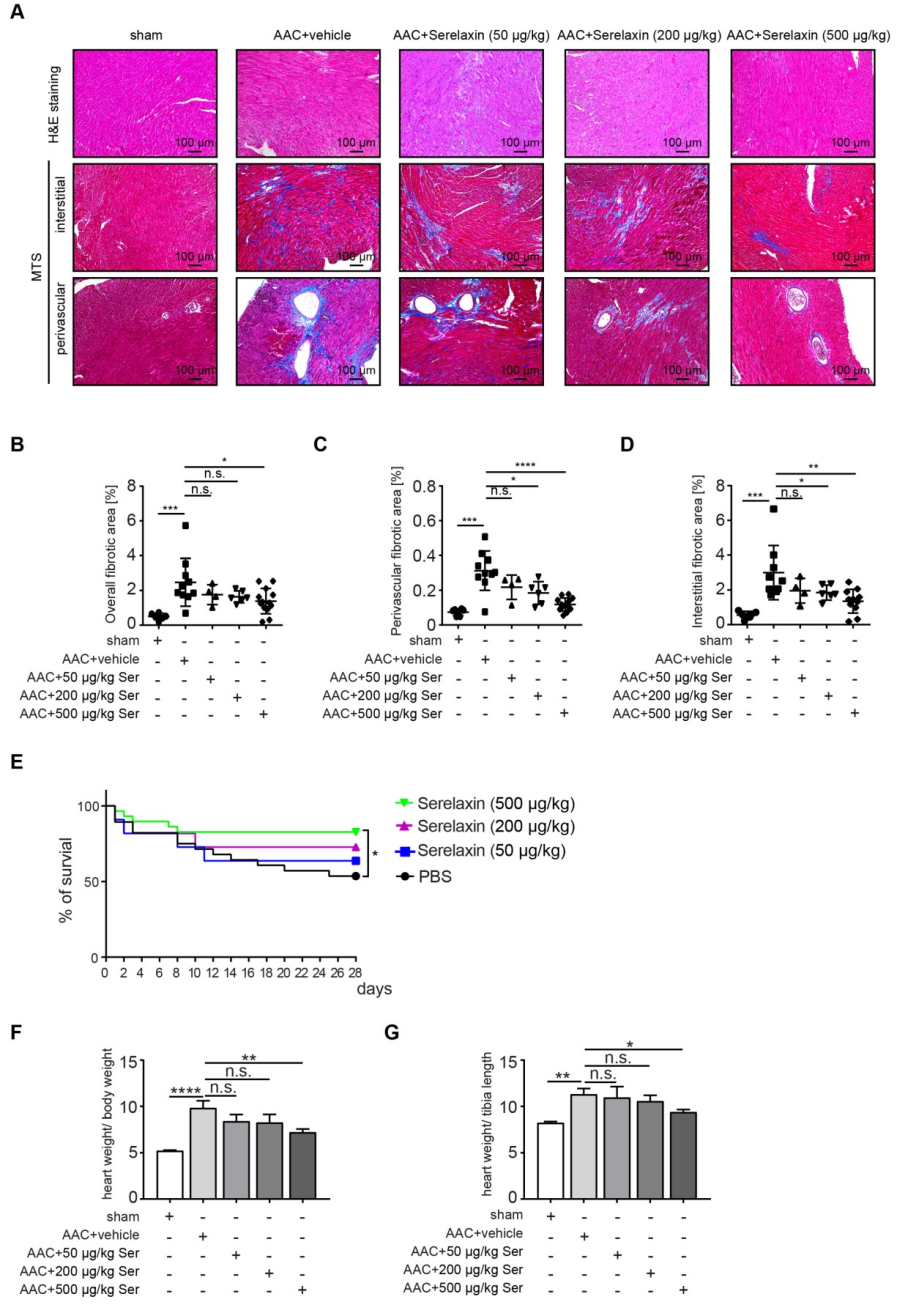
** Serelaxin ameliorates cardiac fibrosis.** (A) HE and Masson's Trichrome Staining microphotography of sham and AAC-operated mouse heart showing the reduction of interstitial and perivascular fibrosis in Serelaxin-treated (500 µg/kg/day) hearts compared to sham and vehicle-treated hearts. (B-D) Graphs showing a significant reduction of fibrosis in Serelaxin-treated hearts. The dot plots represent the percentage of overall, interstitial and perivascular fibrotic area in the sham (n=6), vehicle-treated (n=10) and low (n=4), middle (n=6) and high dose (n=13) Serelaxin-treated AAC mouse hearts. (E) Kaplan-Meier survival curve summarizes survival rates of mice from the AAC operation date until 4 weeks after operation in vehicle-treated (n=28), low (13), middle (13) and high dose Serelaxin-treated (n=29) groups. Administration of high dose Serelaxin substantially increased survival. (F)-(G) Bar graphs showing heart weight related to body weight and tibia length. AAC-operation increased the heart weight compared to sham group, while administration of high but not low and middle dose Serelaxin prevented the increase in heart weight. Student t-test was used for single comparison and one-way ANOVA with Bonferroni post-hoc analysis was used for multiple group comparisons. Overall survival was analyzed by using a Kaplan-Meier survival method with a log rank test to determine statistical differences. Error bars represent mean ± SEM, n.s. no significance, * p<0.05, ** p<0.01, *** p<0.001, **** p<0.0001.

Following clinical studies as well as previous animal studies, which suggested an anti-fibrotic effect of Serelaxin, we aimed to systematically assess the effect of Serelaxin at different doses in two established mouse models of cardiac fibrosis. For this purpose, we induced pressure overload in mice by ascending aortic constriction (AAC) and administered either a vehicle or Serelaxin at 3 different doses at the same time (details are described in the methods section). Echocardiography was performed to verify aortic constriction, and mice were sacrificed after 4 weeks. Quantitative assessment of overall left ventricular fibrotic area by Masson Trichrome staining showed a dose-dependent anti-fibrotic effect of Serelaxin with significant reduction of overall fibrosis by 50% in the high dose Serelaxin group when compared to vehicle (**Figure 1A-B**). We further performed a sub analysis of perivascular fibrosis (defined as fibrotic lesions surrounding vessels of >100 µm) and interstitial fibrosis (defined as all other fibrotic lesions), and demonstrate that while both forms of fibrosis were ameliorated by Serelaxin, the effect of Serelaxin was relatively higher on perivascular as compared to interstitial lesions (**Figure 1A, 1C-D**)**.** Amelioration of cardiac fibrosis was associated with a significantly reduced mortality in mice which had received the high dose of Serelaxin as compared to vehicle treated mice (**Figure 1E**). Analysis of the heart weight in relation to body weight **(Figure 1F)** and tibia length **(Figure 1G)** showed an elevated heart weight in AAC-operated mice compared to sham mice. Administration of high dose Serelaxin significantly prevented the increase in heart weight. AAC-operated hearts showed an enlarged phenotype and echocardiography parameters suggested a dilated left ventricle. Serelaxin could not significantly reverse the heart performance but the heart weight upon high dose administration (**Figure S1**).

As a second independent mouse model, we additionally tested the effect of Serelaxin in an ATII-induced cardiac fibrosis model at two different time points. Mice received 1.5 mg/kg/day ATII and either Serelaxin (500 μg/kg/day) or vehicle for 2 or 4 weeks respectively (**Figure S2A**). Vehicle administered ATII mice developed cardiac fibrosis after 2 weeks and even more pronounced after 4 weeks. However, cardiac fibrosis was significantly reduced in mice which were administered with Serelaxin (**Figure S2B-C**). Both perivascular and interstitial fibrosis were ameliorated by Serelaxin, but the effect of Serelaxin was relatively higher on perivascular as compared to interstitial lesions (**Figure S2D**)**.** This shows that Serelaxin ameliorates cardiac fibrosis in two independent mouse models of cardiac fibrosis.

### Serelaxin alleviates cardiac fibrosis through inhibition of EndMT

**Figure 2:**
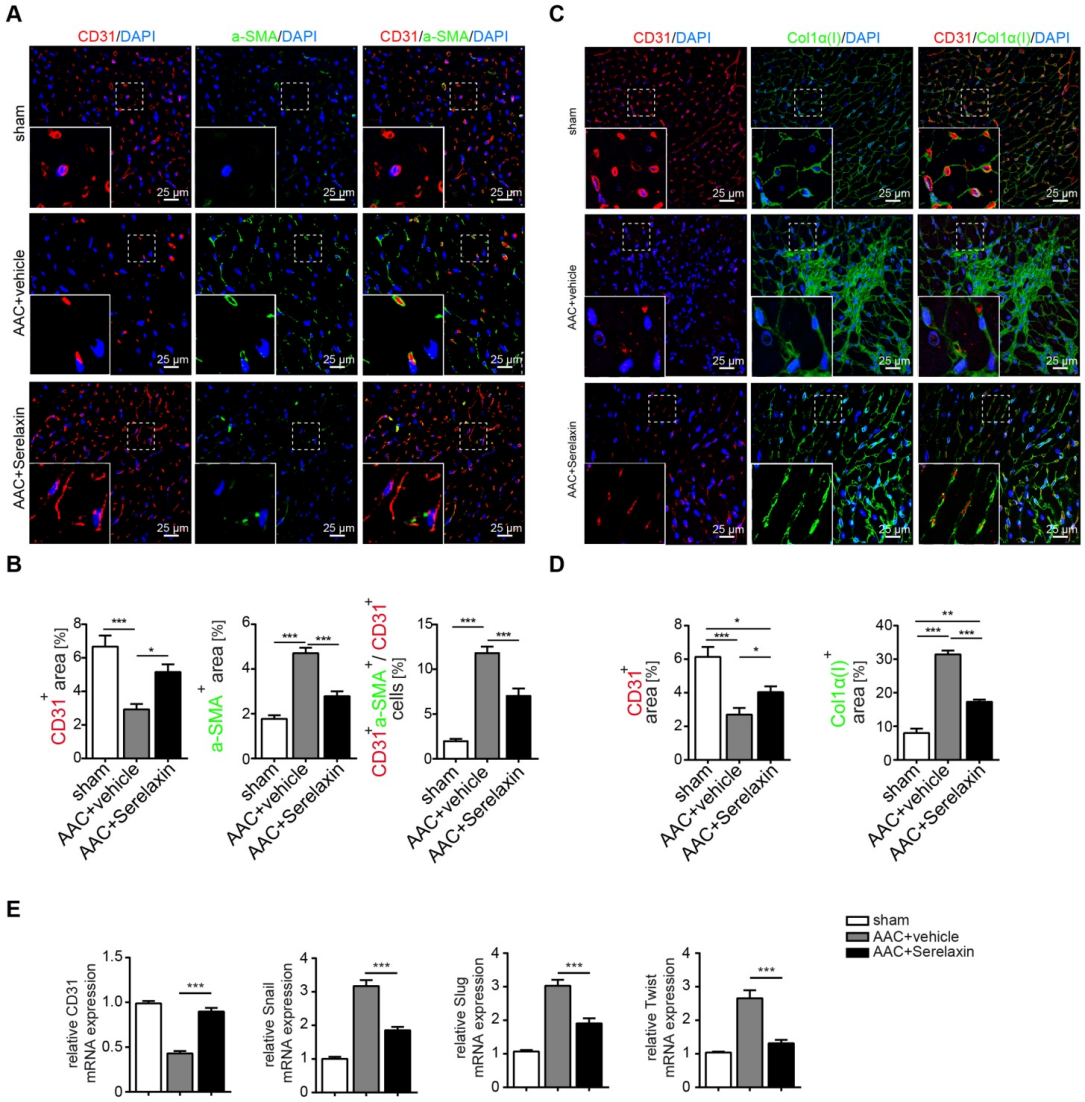
**Serelaxin blocks EndMT in a mouse model of pressure overload.** (A) Immunofluorescence staining of endothelial cell marker CD31, mesenchymal marker α-SMA and DAPI. (B) Quantifications of CD31 and α-SMA positive area and ratio of CD31^+^α-SMA^+^ / CD31^+^ cells. Compared to sham, vehicle-treated hearts showed an increased expression of α-SMA but a decreased protein expression of CD31, while treatment with Serelaxin showed reduced α-SMA and restored CD31 protein expression. Double positive cells significantly increased in AAC-operated animals and were reduced by Serelaxin administration. (C) Immunofluorescence staining of endothelial cell marker CD31, fibrotic marker alpha-1 type I collagen (Col1α(I)) and DAPI. (D) Quantifications of CD31 and Col1α(I) positive area: Col1α(I) expression was upregulated in AAC hearts and is inhibited by Serelaxin. Compared to sham, vehicle-treated hearts showed a decreased protein expression of CD31, while treatment with Serelaxin again restored CD31 expression. (E) qPCR analysis showing the relative mRNA expression level of CD31 and EndMT key regulators Snail, Slug, and Twist in AAC hearts treated with vehicle or Serelaxin. Vehicle-treated AAC-operated hearts showed an increased expression of Snail, Slug and Twist but a decreased expression of CD31, while Serelaxin treatment reduced Snail, Slug and Twist expression and restored CD31 expression. Student t-test was used for single comparison and one-way ANOVA with Bonferroni post-hoc analysis was used for multiple group comparisons. Gene expression and associated error bars represent mean ± SEM, n≥3, n.s. no significance, * p<0.05, ** p<0.01, *** p<0.001.

Because Relaxin acts primarily on vascular cells 42, and since our results indicated a higher effect of Serelaxin on perivascular fibrosis as compared to interstitial fibrosis, we next examined if Serelaxin inhibits EndMT and preserves the endothelial phenotype *in vivo*. We therefore performed immunofluorescence labelling of the endothelial marker CD31 in combination with the mesenchymal markers α-SMA and collagen I (**Figure 2A-D**). Confocal analysis confirmed the abundance of CD31/α-SMA double-positive cells in AAC- challenged hearts indicating the presence of EndMT, as previously described 7,38. In Serelaxin-administered mice, these double positive cells were reduced from 12% to 7% as compared to vehicle-treated mice, suggesting an EndMT-inhibiting effect of Serelaxin (**Figure 2A-B**).

We also analysed the expression of CD31 as well as EndMT transcription factors Snail, Twist and Slug in hearts of vehicle- or Serelaxin-administered mice which underwent AAC surgery (**Figure 2E**). Overall, while AAC is associated with a reduction of CD31-positive cells and a reduction of CD31 expression in the heart, indicating a loss of microvasculature, this reduction was ameliorated in Serelaxin-treated mice, associated with a decrease of collagen I and α-SMA (**Figure 2A-D**). Moreover, the EndMT transcription factors were upregulated in AAC-challenged and ATII-treated hearts as compared to sham animals and vehicle-treated mice respectively. This upregulation was partially blocked in Serelaxin-administered mice (**Figure 2E, Figure S2E**). To exclude that the observed reduction in CD31 is due to apoptosis, we performed an immunohistochemistry staining of cleaved Caspase 3 which is a critical executioner of apoptosis. The staining showed an increased level of cleaved Caspase 3 (3.6%) in AAC-operated hearts compared to sham operated. Serelaxin could not significantly affect cleaved Caspase 3 levels (**Figure S3**). Together with the report by Park et al. who showed that during heart failure endothelial cells contribute to less than 19% of all apoptotic cells of the heart 43, our results suggest an apoptosis-independent effect of Serelaxin.

### Serelaxin inhibits TGFβ1-induced EndMT through RXFP1

**Figure 3:**
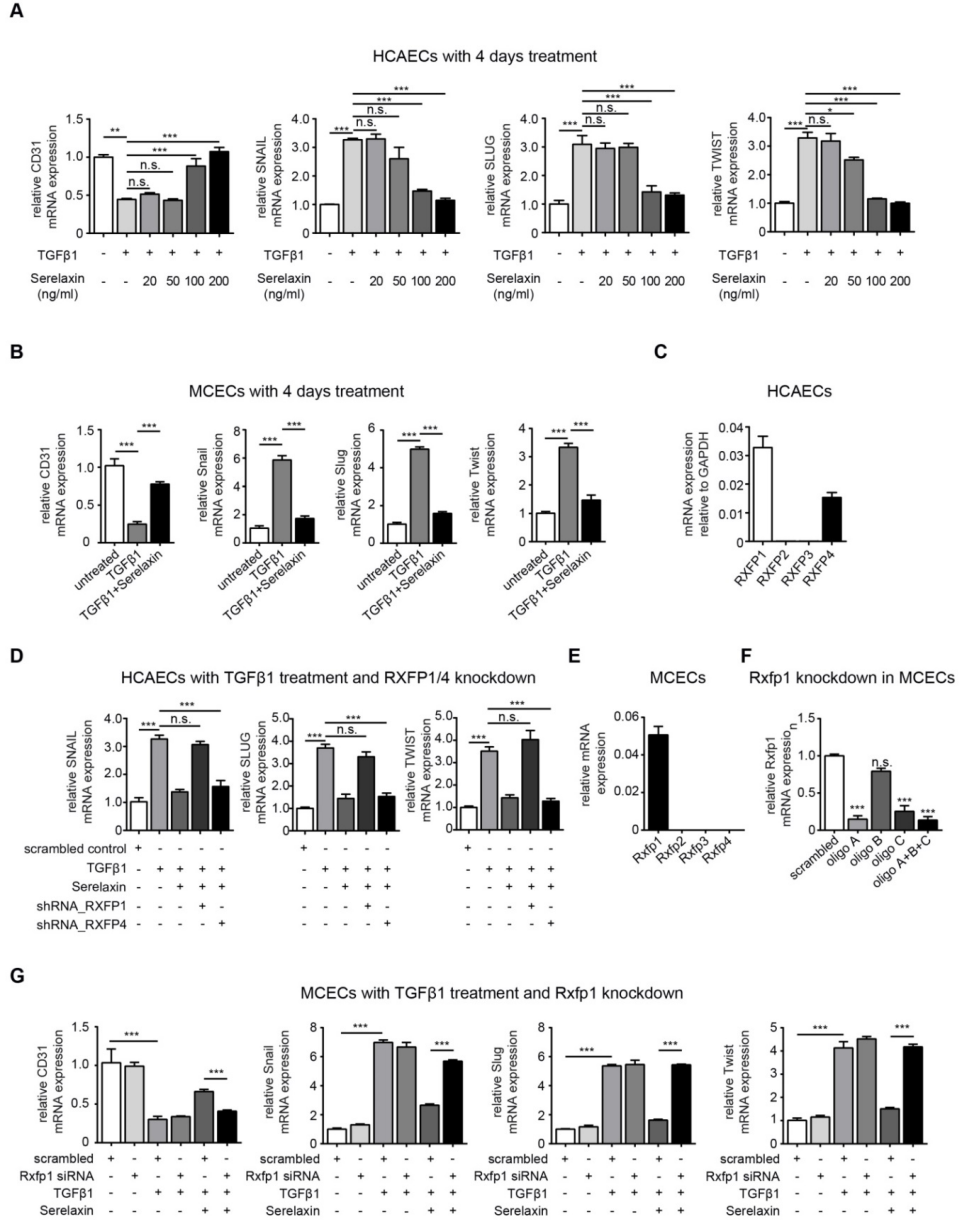
** Serelaxin partially inhibits TGFβ1-induced EndMT in human coronary artery endothelial cells (HCAECs) and mouse cardiac endothelial cells (MCECs) via RXFP1.** (A) qPCR analysis showing the expression of endothelial cells marker CD31 and expression of EndMT key regulators SNAIL, SLUG, and TWIST in TGFβ1-treated HCAECs supplemented with different doses of Serelaxin after 4 days. Cells without any treatment were used as control. Serelaxin treatment significantly rescued expression of CD31 (100 and 200 ng/ml) and decreased expression of SNAIL, SLUG and TWIST (100 and 200 ng/ml). (B) qPCR analysis showing the mRNA expression of EndMT transcriptional factors Snail, Slug and Twist and of endothelial cell marker CD31 in TGFβ1-treated MCECs. Upon TGFβ1 treatment Snail, Slug and Twist expression increased and CD31 expression decreased. Treatment of Serelaxin showed a reduced expression of Snail, Slug and Twist and a restored expression of CD31. (C) qPCR analysis showing the relative mRNA expression level of RXFP1-4 in HCAECs. Among all four genes, only RXFP1 and RXFP4 expression was detectable. (D) qPCR analysis showing the expression of EndMT key regulators SNAIL, SLUG and TWIST in TGFβ1 and Serelaxin-treated HCAECs in combination with knockdown of RXFP1 or RXFP4. Cells without any treatment were used as control. Serelaxin treatment showed a reversal effect on TGFβ1-induced EndMT but not in RXFP1 knockdown cells. (E) qPCR analysis showing the relative mRNA expression level of Rxfp1-4 in MCECs. Among all 4 genes, only Rxfp1 expression was detectable but not the others. (F) qPCR analysis showing the relative mRNA level of Rxfp1 in scrambled and siRNA-mediated Rxfp1 knockdown cells. (G) qPCR analysis showing the expression of EndMT key regulators Snail, Slug and Twist in TGFβ1 and Serelaxin-treated MCECs upon Rxfp1 knockdown. Cells without any treatment were used as control. Serelaxin treatment showed a reversal effect on TGFβ1-induced EndMT but not upon Rxfp1 knockdown. Student t-test was used for single comparison and one-way ANOVA with Bonferroni post-hoc analysis was used for multiple group comparisons. Gene expression and associated error bars represent mean ± SEM, n≥3, n.s. no significance, * p<0.05, ** p<0.01, *** p<0.001.

To gain mechanistic insights into Serelaxin-induced inhibition of EndMT, we used *in vitro* assays of EndMT in human and mouse endothelial cells. We first induced EndMT in HCAECs by TGFβ1 (10 ng/ml), and additionally applied four different concentrations of Serelaxin (ranging from 20 ng/ml to 200 ng/ml). Expression levels of CD31 and of EndMT transcription factors Snail, Twist and Slug were analyzed after 2 and 4 days respectively. Addition of Serelaxin showed a significant restored CD31 expression as well as a decrease in TGFβ1-induced expression of Snail, Twist and Slug at the doses of 100 and 200 ng/ml, indicating inhibition of EndMT (**Figure 3A, Figure S4**). This effect could be observed after 2 days (**Figure S4A**) and was more pronounced after 4 days (**Figure 3A**). Similar to human endothelial cells, addition of 100 ng/ml of Serelaxin was also effective in inhibiting TGFβ1-induced EndMT in MCECs (**Figure 3B**).

In order to identify the receptor type which mediates the EndMT-inhibitory effect of Serelaxin, we analyzed the mRNA expression level of each Relaxin receptor (type 1-4) in HCAECs. We only detected Relaxin receptor 1 (RXFP1) and 4 (RXFP4) to be expressed in these cells (**Figure 3C**). We next performed shRNA-mediated knockdown of RXFP1 and RXFP4 with up to 40% transfection efficiency and successfully decreased expression of both receptors by 80% (**Figure S5A-B**). We next tested the effect of Serelaxin on TGFβ1-induced EndMT in HCAECs upon RXFP1 and RXFP4 knockdown respectively.

Upon knockdown of RXFP4, Serelaxin was still able to reduce the TGFβ1-induced expression of the key EndMT regulators SNAIL, SLUG, and TWIST. However, upon knockdown of RXFP1, the expression levels of the key EndMT regulators were not significantly altered by Serelaxin treatment (**Figure 3D**). Moreover, overexpression of RXFP1 enhanced the inhibitory effect of Serelaxin on EndMT in HCAECs (**Figure S6A**). These results demonstrate that the inhibitory effect of Serelaxin on EndMT is mediated by RXFP1.

In order to gain insights if the anti-fibrotic effect of Serelaxin observed in the mouse models of cardiac fibrosis is also due to RXFP1-mediated inhibition of EndMT, we performed quantitative real time PCR for the different Relaxin receptors in MCECs. In MCECs, only Rxfp1 could be detected (**Figure 3E**).

We silenced Rxfp1 expression by a siRNA technique with three different oligos after which the Rxfp1 expression was reduced by 90% (**Figure 3F**)**.** As in HCAECs, upon knockdown of Rxfp1, the expression levels of CD31 and the key EndMT regulators were not significantly altered by Serelaxin treatment in MCECs (**Figure 3G**). This data suggests that Serelaxin inhibits TGFβ1-induced EndMT through binding to Rxfp1 in both human and mouse endothelial cells.

**Figure 4:**
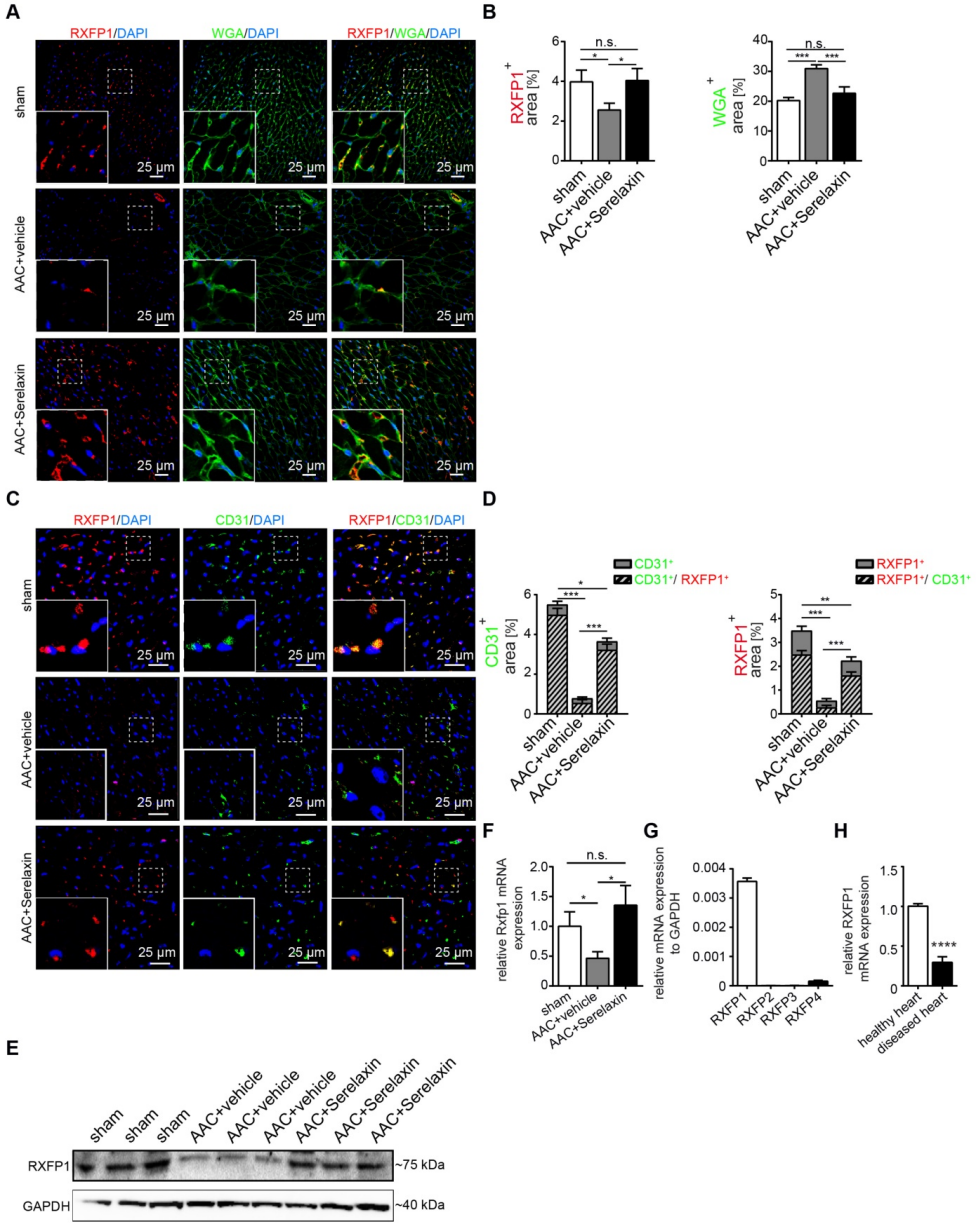
** Serelaxin inhibits EndMT via RXFP1 in vitro and *in vivo*.** (A) Immunofluorescence staining of WGA, RXFP1 and DAPI in sham and AAC-operated hearts treated with vehicle or high dose Serelaxin. (B) Quantification of RXFP1 and WGA positive area showed a decreased RXFP1 and increased WGA protein expression in AAC-operated hearts compared to sham. Upon treatment with Serelaxin, RXFP1 expression was restored and WGA expression decreased. (C) Immunofluorescence staining of endothelial cell marker CD31, RXFP1 and DAPI. (D) Quantifications of CD31 (left panel) and RXFP1 (right panel) positive area. Compared to sham, AAC-operated vehicle-treated hearts showed a decreased expression of RXFP1 and CD31, while treatment with Serelaxin showed increased protein expressions of both CD31 and RXFP1. Each bar shows both the double positive area (streak lines) as well as single positive area (gray). The information on significance refers to the total CD31 (left panel) or RXFP1 (right panel) positive area. (E) Western blot analysis showing protein levels of RXFP1 in sham, AAC-operated and AAC-operated+Serelaxin-treated mouse hearts. Compared to sham, AAC operation reduced RXFP1 expression. Upon administration of Serelaxin, the protein level of RXFP1 was increased. (F) qPCR analysis showing the relative mRNA expression level of Rxfp1 in sham and AAC-operated hearts treated with vehicle or Serelaxin. Rxfp1 expression was decreased in AAC-operated hearts but restored upon Serelaxin treatment. (G) qPCR analysis showing the relative mRNA expression level of RXFP1-4 in human hearts. RXFP1 and RXFP4 expression were detected whereas RXFP1 was mainly expressed. (H) qPCR analysis showing a reduced relative mRNA expression level of RXFP1 in diseased human hearts compared to healthy control hearts. Student t-test was used for single comparison and one-way ANOVA with Bonferroni post-hoc analysis was used for multiple group comparisons. Gene expression and associated error bars, representing mean ± SEM, n≥3, n.s. no significance, * p<0.05, ** p<0.01, *** p<0.001, **** p<0.0001.

We next analyzed RXFP1 protein expression *in vivo* by immunofluorescence staining (**Figure 4A-D**) and Western blot (**Figure 4E**) and mRNA expression by qPCR (**Figure 4F**) in the AAC-induced cardiac fibrosis model. In sham control mice, RXFP1 is largely colocalized (91%) with CD31 expression (**Figure 4D**). Interestingly, the expression level of Rxfp1 was decreased in diseased hearts as compared to healthy controls, but upregulated upon Serelaxin treatment (**Figure 4D-F**). In human heart tissue from healthy controls, RXFP1 is also the most abundant among all four receptors (**Figure 4G**), and is significantly decreased in tissue of patients with end stage heart failure (obtained at the time of heart transplantation) (**Figure 4H**).

### Serelaxin inhibits TGFβ1-induced EndMT through preservation of Notch signaling in endothelial cells

**Figure 5:**
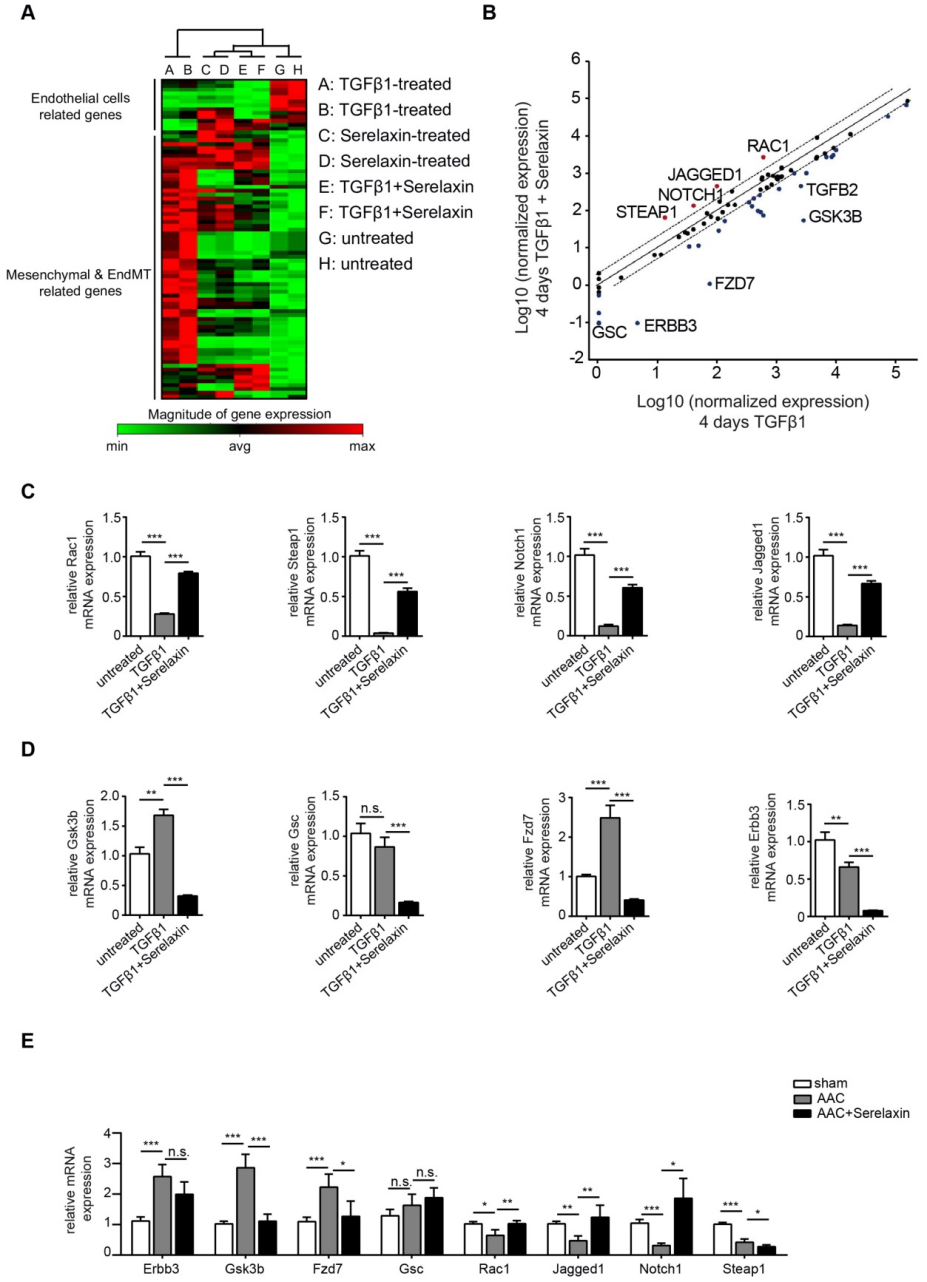
** Serelaxin promotes the preservation of endothelial cell properties in both *in vivo* and *in vitro* by regulating candidate genes.** (A) Heat map and (B) scatter plot show the genes with altered mRNA expression level in only TGFβ1-treated (x-axis) or in both TGFβ1- and Serelaxin-treated (y-axis) HCAECs. STEAP1, NOTCH1, JAGGED1, RAC1, DSP, GSC, ERBB3, FZD7, GSK3B and TGFB2 were significantly regulated by Serelaxin treatment, which is shown by separation from dot lines (cut-off by 4 folds). (C-D) qPCR analysis showing the expression of candidate genes, which were identified from qPCR array in TGFβ1- and Serelaxin-treated MCECs. Cells without any treatment were used as control. (E) qPCR analysis of the panel of candidate genes *in vivo* in sham and AAC-operated mice treated with vehicle or Serelaxin. Serelaxin significantly rescued the effects of AAC on all genes except for Gsc and Steap1. Student t-test was used for single comparison and one-way ANOVA with Bonferroni post-hoc analysis was used for multiple group comparisons. Gene expression and associated error bars, representing mean ± SEM n≥3, n.s. no significance, * p<0.05, ** p<0.01, *** p<0.001.

In order to gain insights into the downstream signaling pathways affected by Serelaxin treatment, we performed an EndMT qPCR array analysis (containing 84 key genes that either change their expression during the EndMT process or regulate those gene expression changes). This assay was performed in HCAECs after 4 days with either no treatment, TGFβ1 treatment alone, Serelaxin treatment alone or both TGFβ1 and Serelaxin treatment. The overall gene expression profile of TGFβ1/Serelaxin combination treatment was closest to that of Serelaxin-treated samples and then to the untreated sample group in hierarchy analysis (**Figure 5A**).

We then compared the differentially expressed genes between TGFβ1 treatment alone and TGFβ1/Serelaxin combination treatment (**Figure 5B**) and between Serelaxin treatment alone and TGFβ1 treatment alone (**Figure S7**). Four genes (STEAP1, NOTCH1, JAGGED1 and RAC1) were selectively upregulated in the Serelaxin treatment alone and TGFβ1/Serelaxin combination group and six genes (GSC, ERBB3, FZD7, GSK3B and TGFB2) in the TGFβ1 group. Notably, Serelaxin ameliorated the effects of TGFβ1 on these genes in HCAECs.

**Figure 6:**
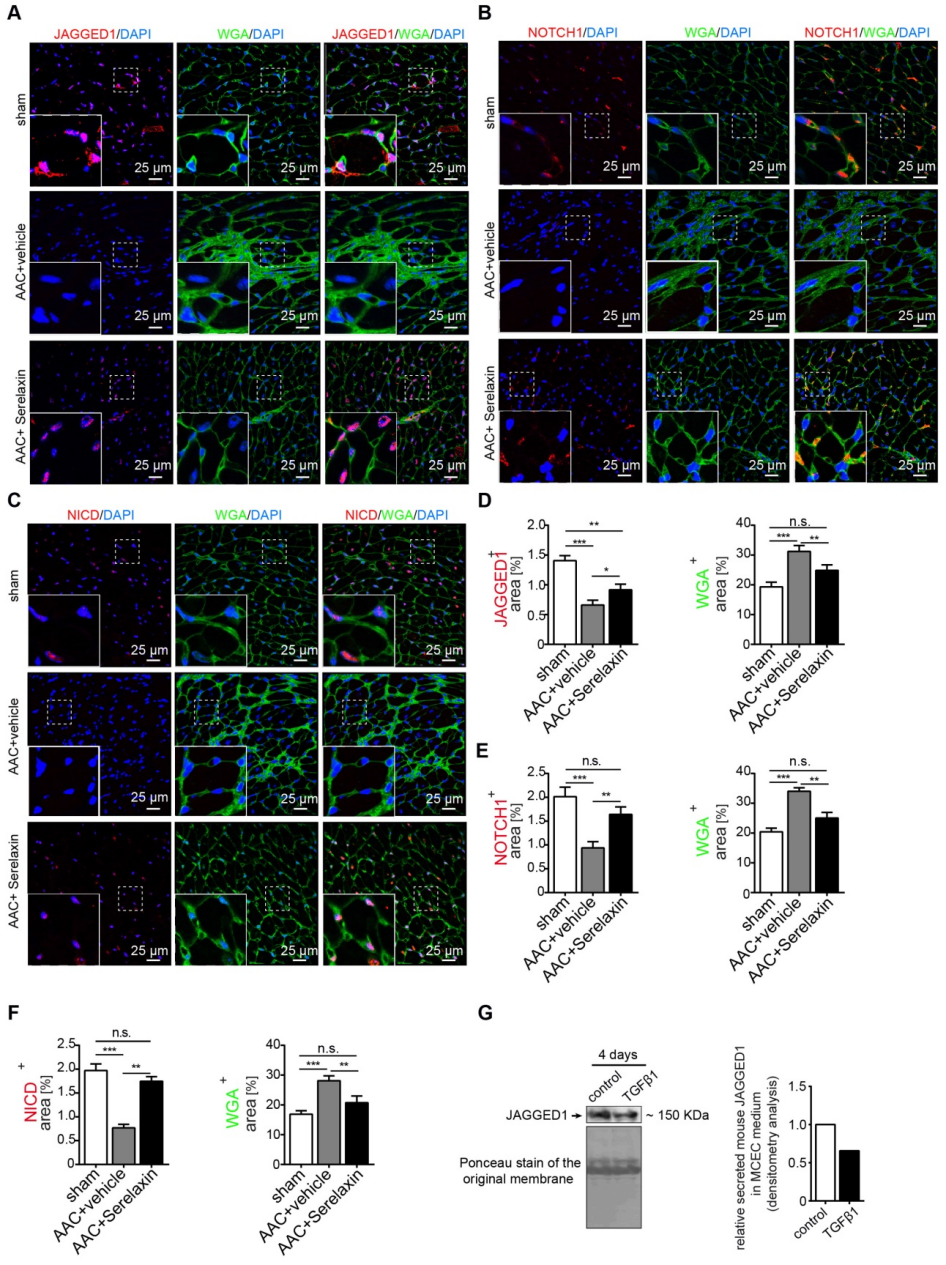
** Serelaxin rescues the Notch1 pathway in AAC-operated hearts.** Immunofluorescence staining of (A) Jagged1, (B) Notch1 and (C) NICD in combination with WGA and DAPI in sham and AAC-operated hearts treated with vehicle or Serelaxin. Quantifications showed that protein expressions of (D) Jagged1, (E) Notch1 and (F) NICD were downregulated in AAC-operated hearts and could be restored by Serelaxin. (G) Western blot analysis shows the amount of the soluble form of Jagged1 in the medium of TGFβ1-treated MCECs. Ponceau-S stained membrane picture indicates that an equal amount of total precipitated protein was loaded for both the control and TGFβ1-treated MCECs. Soluble Jagged1 was reduced in TGFβ1-treated MCECs as quantified by densitometry analysis (right panel). Student t-test was used for single comparison and one-way ANOVA with Bonferroni post-hoc analysis was used for multiple group comparisons. Gene expression and associated error bars, representing mean ± SEM, n≥3, n.s. no significance, * p<0.05, ** p<0.01, *** p<0.001.

To validate those candidate genes in mice, we analyzed their expression in MCECs and in mouse heart tissue. In Serelaxin supplemented TGFβ1-treated MCECs, Rac1, Steap1, Notch1 and Jagged1 were all significantly upregulated whereas Gsk3b, Gsc, Fzd7 and Erbb3 were significantly decreased as compared with TGFβ1-treated cells (**Figure 5C-D**), which is in line with the experiments performed in HCAECs. In hearts of AAC-challenged mice which received Serelaxin, three of the upregulated genes (Rac1, Jagged1 and Notch1) and three of the downregulated genes (Erbb3, Gsk3b and Fzd7) could be validated (**Figure 5E**). Unlike Erbb3, Steap1 (which are reduced both in endothelial cells upon TGFβ1 *in vitro*, and also in whole AAC hearts *in vivo*) is rescued by Serelaxin only *in vitro* but not *in vivo* (where Serelaxin even further decreases Steap1 expression). This discrepancy is likely due to different regulation of Steap1 in different cell types. Because out of these genes Notch1 and its ligand Jagged1 (both upregulated by Serelaxin treatment) have well-known protective roles for maintaining the endothelial phenotype, we focused on studying the effect of Serelaxin on Notch signaling. Immunofluorescence staining of AAC-challenged hearts revealed fewer Jagged1, Notch1 and NICD-positive cells (indicating reduced Notch1 intracellular signaling) as compared to sham operated mice, especially within fibrotic areas (as indicated by wheat germ agglutinin staining, WGA, **Figure 6A-C**).

In Serelaxin-administered mice, Jagged1, Notch1 and NICD-positive areas were all increased as compared to vehicle-treated mice (**Figure 6D-F**), indicating that the effect of Serelaxin which we observed during EndMT *in vitro* also occurs during fibrogenesis *in vivo*. To test if this effect of Serelaxin is similarly mediated by Rxfp1, we performed siRNA knockdown of Rxfp1 in MCECs. Afterwards we induced EndMT by TGFβ1 and tested the effect of Serelaxin with respect to Notch1 and Jagged1 expression (**Figure S6B-D**). While Serelaxin rescued Notch1 and Jagged1 expression in TGFβ1-induced EndMT, this effect was gone upon knockdown of Rxfp1. In order to further elucidate the link between Serelaxin, Rxfp1 and Notch1, we have additionally transfected TGFβ1-treated MCECs with a NICD overexpression construct (to mimic Notch1 activation), which resulted in reduction of EndMT marker genes (**Figure S6E**). On the other hand, supplementation of DAPT (an indirect Notch inhibitor) to Serelaxin + TGFβ1-treated MCECs lead to a significantly decreased EndMT-inhibitory effect of Serelaxin as compared to supplementation of DMSO as a control (**Figure S6F**). This data implies that Serelaxin ameliorates TGFβ1-induced EndMT, at least in part, via activation of the Notch pathway.

Because Jagged1 mediates Notch1 activation only after being secreted, we also measured Jagged1 secretion from MCECs. We therefore collected medium from TGFβ1-treated and control MCECs and precipitated all soluble proteins by acetone. Western blot analysis confirmed a significant decrease of secreted Jagged1 soluble protein in MCECs upon TGFβ1 treatment in comparison to untreated MCECs (**Figure 6G**). To sum up, our results suggest that the Notch1 signaling pathway is partially inhibited in AAC-operated hearts and TGFβ1-treated MCECs. These effects were attenuated by supplementation of Serelaxin.

### Serelaxin rescues Rxfp1 expression by modulating histone modifications via inhibition of TGFβ pathway

Overall, the results thus far imply that Serelaxin exerts its anti-fibrotic effect at least in part by inhibition of EndMT via the Notch1 signaling pathway, which is mediated by Rxfp1. However, we have observed, that in AAC-challenged mouse hearts** (Figure 4E-F)**, human diseased hearts (**Figure 4 H**) as well as in TGFβ1-treated endothelial cells (**Figure S6B**); RXFP1 is downregulated as compared to healthy hearts or native endothelial cells. In mice which were administered with Serelaxin on the other hand, this receptor downregulation was rescued (**Figure 4E-F**). In this respect, it has been shown that TGFβ1 induces epigenetic silencing of gene expression via methylation of CpG islands within the promoter as well as via histone modifications. We therefore aimed to investigate if the silencing of Rxfp1 could be due to one of these epigenetic mechanisms and if Serelaxin is able to inhibit such modifications. However, within 5 kb upstream of the transcription start site (TSS) of murine Rxfp1 we have not found a CpG island, indicating that silencing of Rxfp1 is not mediated by promoter hypermethylation.

**Figure 7:**
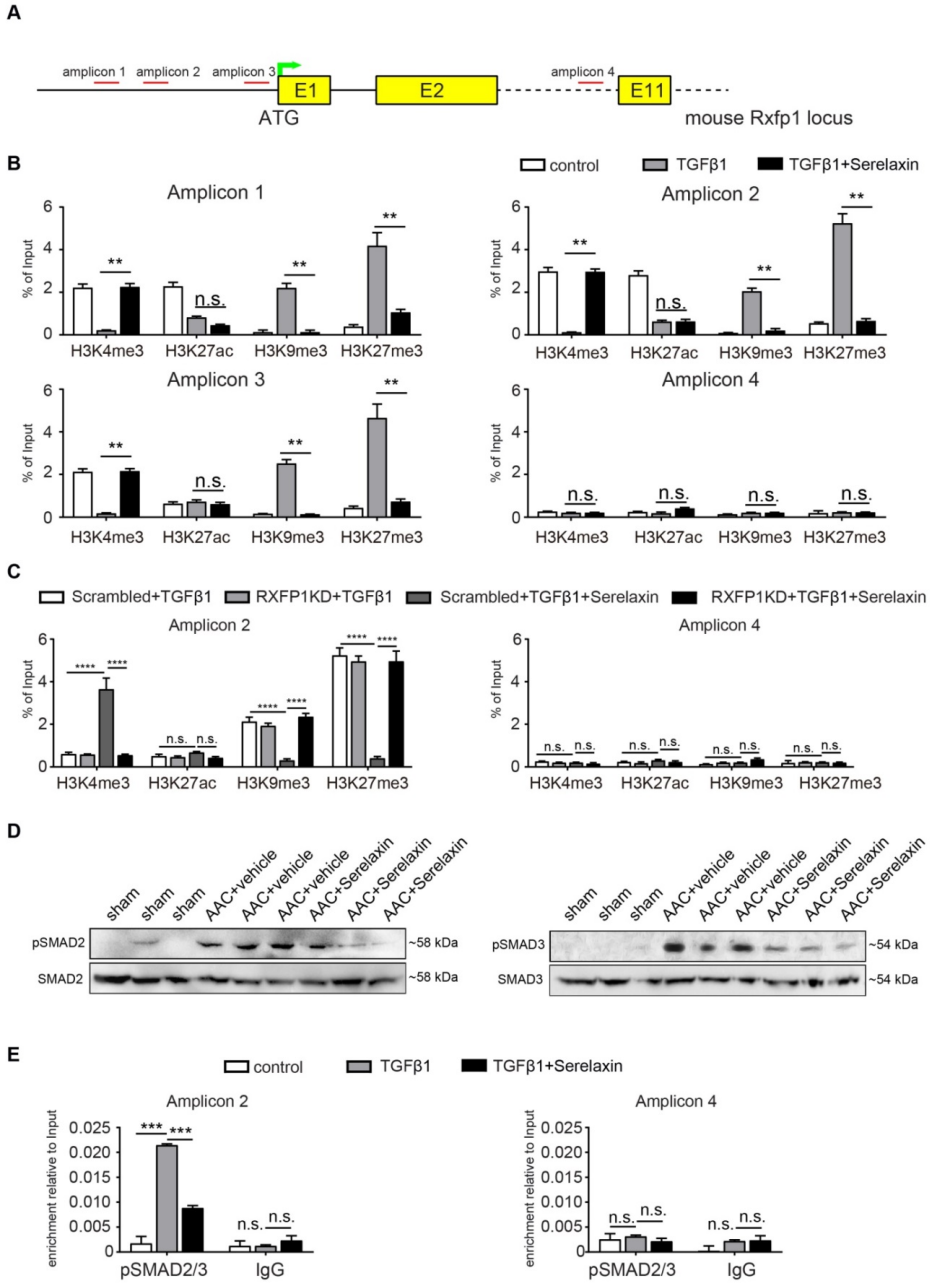
** Serelaxin reactivates Rxfp1 expression via histone modifications.** (A) Schematic representing the mouse Rxfp1 locus with locations of CHIP-qPCR primers. (B) qPCR analysis showing the enrichment of 4 different histone modification marks (active: H3K4me3 and H3K27ac, repressive: H3K9me3, H3K27me3) in the Rxfp1 promoter region (Amplicon 1-3) and intron 10 (Amplicon 4). The enrichment of TGFβ1-influenced H3K4me3, H3K9me3 and H3K27me3 marks were significantly compromised by Serelaxin if performed with amplicons 1-3 targeting the promoter region but not with amplicon 4 targeting intron10. (C) ChIP-qPCR analysis showing the enrichment of histone modification marks in the *Rxfp1* promoter region after RXFP1 knockdown. Upon RXFP1 knockdown, treatment with Serelaxin did not significantly increase the activating modifications or decrease the repressive modification marks. (D) Western blot analysis showing protein levels of pSMAD2 and pSMAD3 in sham, AAC-operated and AAC-operated+Serelaxin-treated mouse hearts, total SMAD2 and SMAD3 were used as protein loading controls. AAC-operated hearts showed increased levels of pSMAD2 and pSMAD3 compared to sham. Upon treatment with Serelaxin these protein levels were significantly reduced. (E) qPCR analysis showing the enrichment of pSMAD2/3 in the *Rxfp1* promoter region (Amplicon 2) and intron 10 (Amplicon 4). The enrichment of TGFβ1-induced pSMAD2/3 was significantly compromised by Serelaxin. The qPCR analysis with primer targeting intron10 did not show any significant differences between these groups. Student t-test was used for single comparison and one-way ANOVA with Bonferroni post-hoc analysis was used for multiple group comparisons. Gene expression and associated error bars, representing mean ± SEM, n≥3, n.s. no significance, ** p<0.01, *** p<0.001, **** p<0.0001.

In order to identify potential histone modifications facilitated by TGFβ1 and/or Serelaxin, we performed ChIP analysis of gene specific histone modification profiles (activating: H3K4me3, H3K27ac; and repressive: H3K9me3, H3K27me3) at the promoter region (1kb upstream of TSS) of Rxfp1 (**Figure 7A**).

Real-time PCR showed that the promoter region of Rxfp1 was enriched for the activating modifications H3K4me3 and H3K27ac and depleted for the repressive modifications H3K9me3 and H3K27me3 in untreated cells, indicating a transcriptionally active chromatin (**Figure 7B**). In contrast, after TGFβ1 treatment, the repressive modifications were increased whereas the activating modifications were reduced (**Figure 7B**). After supplementation with Serelaxin, the activating modifications could be significantly increased and repressive modification marks were significantly decreased (**Figure 7B**). The qPCR analysis with a pair of primer targeting intron 10 did not show any significant differences among all the histone marks. To further explore the dependence of histone modification changes on Rxfp1 expression, we repeated ChIP analysis with Rxfp1-knockdown MCECs and found that Serelaxin was no longer able to increase the activating modifications nor to decrease the repressive modifications (**Figure 7C**). Collectively, these results suggest that restored Rxpf1 expression upon Serelaxin treatment is mediated at least in part through a histone modification regulatory mechanism facilitated by Serelaxin on the remaining Rxfp1 expression.

Since it is known that Relaxin inhibits the TGFβ pathway 44-46, we hypothesized that activation of RXFP1 expression by Serelaxin might be through blocking the TGFβ pathway. We performed Western blot analysis of pSMAD2 and pSMAD3 in sham, AAC-operated and AAC-operated+Serelaxin-treated mouse hearts and showed an increase of pSMAD2 and pSMAD3 protein levels in AAC-operated animals compared to sham. Upon administration of Serelaxin, the protein levels of pSMAD2 and pSMAD3 were decreased (**Figure 7D**). pSMAD2/3 are transcription factors which bind to DNA, regulate the target gene expression and are known to mediate histone marks (H3K27me3, H3K9me3, H3K4me3, H3K27ac). In order to further elucidate the link between Serelaxin, pSMAD2/3 and RXFP1 expression, we performed a pSMAD2/3 immunoprecipitation experiment at the promoter region of *Rxfp1* and showed that the enrichment of pSMAD2/3 at the *Rxfp1* promoter region was significantly compromised by treatment with TGFβ1+Serelaxin when compared to TGFβ1 treatment alone (**Figure 7E**). The qPCR analysis with the primer targeting intron 10 did not show any significant differences between these groups.

**Figure 8:**
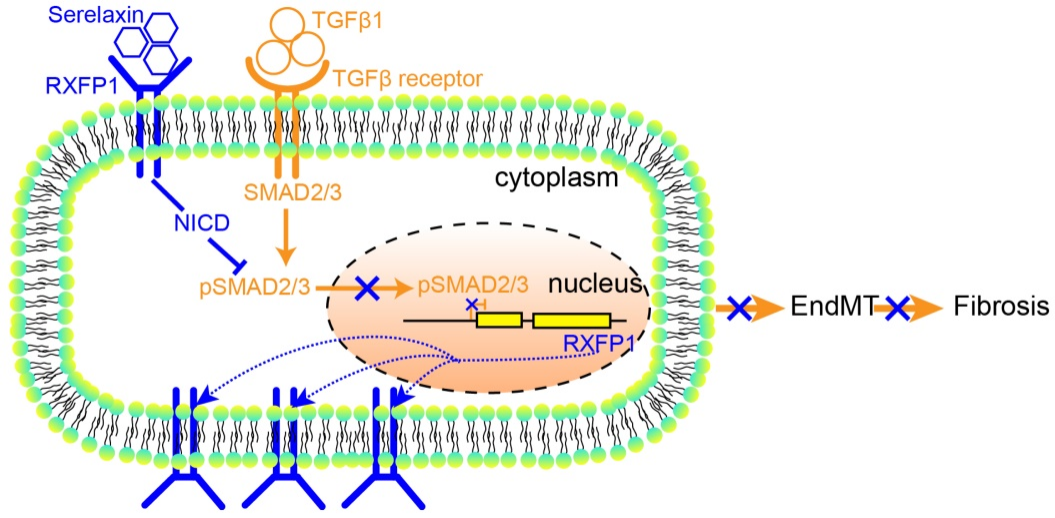
A proposed model depicting the molecular mechanism by which Serelaxin activates Notch signaling pathway to inhibit TGFβ-induced SMAD2/3 phosphorylation to restore the RXFP1 expression via histone modifications.

These results suggest that Serelaxin initiates a positive feedback loop: Serelaxin induces Rxfp1 expression by partially inhibiting TGFβ-induced histone modifications at the* Rxfp1* promoter (**Figure 8**).

## Discussion

This study demonstrates an anti-fibrotic effect of Serelaxin in two independent mouse models of pressure overload, AAC and the ATII infusion via osmotic minipumps. We also showed that the anti-fibrotic effect is associated with reduced mortality in mice. We further demonstrate that the anti-fibrotic effect of Serelaxin is at least in part due to inhibition of EndMT via activation of Notch1 signaling, and that this effect is mediated by the Relaxin receptor Rxfp1. Finally, we provide data, which suggest that Serelaxin is able to rescue low Rxfp1 levels in diseased hearts by blocking the TGFβ pathway and consequently mediating Rxfp1 gene histone modifications.

With respect to controversial reports on the relevance of EndMT in pressure overload models, it is important to note that in this study we used constriction of the ascending aorta (“AAC”, a more pronounced model of pressure overload, where EndMT has been previously demonstrated 38) but not constriction of the transverse aorta (“TAC” a milder model, where EndMT has not been reported 47). Interestingly, we found downregulation of Notch1 signaling (which has been linked to increased EndMT by several studies 30,31) 4 weeks after AAC accordingly, whereas increased Notch1 signaling has been reported one week after TAC (where no EndMT is present). As a second model we used Angiotensin II infusion via osmotic minipumps, which yielded similar results. We are aware however, that this model (while in general well-accepted to induce pressure-overload and cardiac fibrosis) also exerts direct pro-fibrotic stimuli to the heart, e.g. via direct activation of the TGFβ1/Smad signaling pathway in cardiac fibroblasts 48. Thus, an additional influence of Serelaxin on these stimuli cannot be excluded in this model.

Nevertheless, in both animal models, Serelaxin showed consistent results in the sense that the reduction of perivascular fibrosis was more significant than interstitial fibrosis, which guided our focus on studying the effect of Serelaxin with respect to EndMT. Since perivascular fibrosis is also a contributor to reduction of myocardial and arterial compliance 49, the therapeutic effect of Serelaxin on perivascular fibrosis observed by us is in line with a recent study which showed a vascular-protective effect of Serelaxin in an ATII mouse model 50.

This study is in accordance with several other studies which reported an anti-fibrotic effect of Serelaxin in various organs including the heart 51-53.

While most other studies focused on studying effects of Serelaxin on fibroblasts, in our study Serelaxin showed a microvasculature stabilizing and profound EndMT-inhibitory effect on endothelial cells. We could prove that Serelaxin is indeed able to ameliorate fibrosis, at least in part, via inhibition of EndMT. Both inhibition of fibroblast to myofibroblasts activation 54 and inhibition of EndMT likely affect fibrosis in parallel and synergistically.

In line with this, Wu et al. recently showed that Serelaxin inhibits TGFβ1/Smad2/3 signaling pathway through inhibiting the expression of ALK5 in TGFβ1-treated cardiac fibroblasts 55.

That EndMT contributes to fibrogenesis was first reported in cardiac fibrosis in a model of pressure overload, but in subsequent studies it was shown that EndMT is also relevant in fibrosis of other organs including the kidney, the lung and the intestinal tract 33,37,56. Besides, EndMT contributes to cancer-associated fibroblasts in the tumor stroma, where it is associated with metastasis 36. It could therefore be speculated that Serelaxin has an effect on fibrosis in all organs where EndMT is involved and potentially, that it affects progression of tumors associated with EndMT.

Several tumor studies describe that Relaxin promotes matrix invasion by affecting the Wnt/β-Catenin and GSK3β pathway 57-59. These studies found that supplementation of Relaxin increased protein levels of phosphorylated GSK3β but not of total GSK3β. We have studied mRNA of total GSK3β (which we find decreased in cardiac endothelial and whole heart tissue upon Serelaxin). In general, this finding supports Thanasupawat et al., who summarized in 2019 that the positive or negative effect of Relaxin on cells depends on the cell model chosen, the exposure time, and on the Relaxin concentration used 60.

Our study identified Notch1 signaling to be reactivated by Serelaxin both *in vitro* and *in vivo*. Because Notch1 signaling is able to both induce and inhibit EndMT (depending on the context), we performed NICD overexpression to study the effect of Notch1 in the context of adult coronary artery endothelial cells. Our results suggest that Notch1 protects the endothelial phenotype in this context. Our study is supported by a study in a rat model of heart failure, where a link between Relaxin and Notch1 signaling has also been observed 30. The effect of TGFβ1 on Notch1 is equally context-dependent: a positive regulatory role of TGFβ1 in activating Notch signaling pathway has been reported in other cells. Blokzijl et al. showed a cross-talk between the Notch1 and TGFβ signaling pathways in mouse neural stem cells and mouse myoblasts 61. Upon TGFβ1 treatment, Hes1 (the target for Notch pathway) expression was increased. Hajdu et al. also showed an increased HES1 and TIEG expression in human non-Hodgkin B-cell lymphoma cell lines 62. Both studies focused on the effect of TGFβ1 treatment on Notch1 target gene expression rather than on Notch1 expression itself. In contrast to these studies, Wu et al. showed (similar to our results) a reduced Notch1 target gene expression by TGFβ1 in adult human Mueller stem cells 63. Interestingly, a reduction of Notch1 by TGFβ1 in cardiac fibroblast has also been reported by Sassoli et al 24. The discrepancy between these various studies underlines the cell-type specific regulation of Notch1 signaling, and potential cell-type specific effects of Serelaxin.

Furthermore, our study is the first to identify Rxfp1 to mediate the anti-EndMT effect of Serelaxin. We identified this receptor in *in vitro* studies of EndMT, and found this receptor to be downregulated in diseased hearts as compared to healthy hearts *in vivo* accordingly. The fact that administration of Serelaxin to mice which had undergone AAC was able to rescue expression of this receptor to the level of sham operated animals is potentially one of the most important and novel findings of this study. Our data suggests that in diseased hearts, Rxfp1 is downregulated through histone modifications and that Serelaxin is able to influence these chromatin modifications in a favorable way. This study is the first to report that Serelaxin affects histone modifications by an anti-TGFβ-SMAD2/3 cascade (**Figure 8**). The recent phase III trial (RELAX-AHF 2), testing short-term intravenous Serelaxin administration for acute decompensated heart failure, did not show a reduced cardiovascular mortality after 6 months nor reduced worsening of heart failure after 5 days and therefore failed. However, our results suggest that Serelaxin may have long-term beneficial effects in chronic heart failure through its anti-fibrotic and epigenetic gene re-activating effects.

## Supplementary Material

Supplementary figures.

## Figures and Tables

**Table 1:** Antibodies used in Immunofluorescence staining

Antibody	Dilution	Company	Catalog No.
Anti-Mouse wheat germ agglutinin Alexa Fluor 488 conjugated	1:1000	Life technologies	W11261
Rabbit Anti-Mouse RXFP1	1:50	Santa Cruz	SC50528
Rabbit Anti-Mouse αSMA	1:100	Abcam	Ab32575
Goat Anti-Mouse CD31	1:50	Santa Cruz	SC1306
Rabbit Anti-Mouse Col1α(I)	1:200	Abcam	Ab34710
Rabbit Anti-Mouse Notch1	1:100	Abcam	Ab8925
Rabbit Anti-Mouse Jagged1	1:50	Santa Cruz	SC8303
Rabbit Anti-Mouse NICD	1:50	Abcam	Ab52627
Alexa Fluor 568 donkey anti rabbit	1:200	Life technologies	A10042
Alexa Fluor 568 donkey anti mouse	1:200	Life technologies	A10037
Alexa Fluor 568 donkey anti goat	1:200	Life technologies	A11057
Alexa Fluor 488 donkey anti rabbit	1:200	Life technologies	R37118

**Table 2:** Antibodies used in ChIP-qPCR

Antibody	Dilution	Company	Catalog No.
H3K4me3	1:400	ActiveMotif	39159
H3K9me3	1:400	ActiveMotif	39765
H3K27me3	1:400	ActiveMotif	39155
H3K27ac	1:400	ActiveMotif	39133
pSMAD2/3	1:300	Cell Signaling	5678

**Table 3:** Primers used in ChIP-qPCR assay for Rxfp1

ChIP-qPCR assay for Rxfp1	
Amplicon	Sequence
1	F: TGCTCAACTTTCCAAACAGA R: ATGCTTTTGTGGCACAGCTA
2	F: CCATGCTTGGGATTTACCTC R: TGCTCTGACAAAGCCTTCAC
3	F: ATGAGGGAGGGACACAGAGA R: ACAGCTCACAGTGGTTGTGC
4	F: TGGTGTGGGGATTGAACTCA R: GACTTCATGCATGTGGAGGC

**Table 4:** Primers used in qPCR assay for gene expression analysis

Species	Gene	Sequence	Supplier
Mouse	CD31	F: CCAAAGCCAGTAGCATCATGGTC R: GGATGGTGAAGTTGGCTACAGG	Primer design
	Erbb3	F: TCACACTCAGCCCGTTTAGA R: AGGTGCTGGGTTTCCTTCTC	Origene
	Fzd7	F: TCAGCTGGAGGAAAAAGACG R: GTGCTGGACGCGGAGAGT	Origene
	Gapdh	undisclosed	Primer design
	Gsc	F: CATGTAGGGCAGCATCTGGT R: CAGCAGTGCTCCTGCGTC	Origene
	Gsk3b	F: GTGGTTACCTTGCTGCCATC R: GACCGAGAACCACCTCCTTT	Origene
	Jagged1	F: AACCTGTAACATAGCCCGAAAC R: GTAAAGGACTCGCCGTTGAC	Primer design
	Notch1	F: TGCCCGTTCTTGAAATGTAGG R: GGCAGTGTCTTTCCCCAGA	Primer design
	Rac1	F: TCTCCAGGAAATGCATTGGT R: AGATGCAGGCCATCAAGTGT	Origene
	Rln1	F: GGCAACCATCATTACCAGAGC R: TCCAAGCCTAAGTATTTTAATTCTGAA	Primer design
	Rxfp1	F: ATTTCTCTCTGCTGTGCTGACT R: CGGCTGTGCGTGCTTATTG	Primer design
	Rxfp2	F: ACGAACTCCACCTTCCTAACG R: AAAATGTCTTCTCTGGAACAAAACC	Primer design
	Rxfp3	F: AACCCGATCCTCTACTGCTTAG R: GCATGTTGGTGAGCGAAGG	Primer design
	Rxfp4	F: AGGTAACTGTGGTCAGCGTGTG R: CCAGTGGAAGTCCATTGCTGAC	Origene
	Slug	F: CGCTCCTTCCTGGTCAAGA R: AGGTATAGGGTAACTTTCATAGAGATA	Primer design
	Snail	F: GTGCCCACCTCCAAACCC R: AAGGACATGCGGGAGAAGG	Primer design
	Steap1	F: CAAACCCAGAACAACTTTGGA R: TCGTCTCTCCCGAGTCCTTA	Origene
	Twist	F: TGATAGAAGTCTGAACACTCGTTTG R: GGCTGATTGGCAAGACCTCT	Primer design
	CD31	F: AAGGAACAGGAGGGAGAGTATTA R: GTATTTTGCTTCTGGGGACACT	Primer design
	GAPDH	undisclosed	Primer design
	RLN1	F: GGCAACCATCATTACCAGAGC R: TCCAAGCCTAAGTATTTTAATTCTGAA	Primer design
	RLN2	F: GCTCCTCAGACACCTAGACC R: CTGTGGCAAATTAGCAACAAATTC	Primer design
	RXFP1	F: GCTGTATGCCATGTCAATCATT R: TCTCCACGAAACTTTAGGTCAA	Primer design
	RXFP2	F: GATCACTCCTTCATGCCAAAAAG R: TGTCACCACAGTTCTCTTCGT	Primer design
	RXFP3	F: ACCAAATCAGTGACCATCGTT R: GCGTTGAACTTGATGAGGATG	Primer design
	RXFP4	F: CCTGTCACTACTTGCTTGGCAC R: TCAACCGCAGATCCCTGAAGGT	Origene
	SLUG	F: ACTCCGAAGCCAAATGACAA R: CTCTCTCTGTGGGTGTGTGT	Primer design
	SNAIL	F: GGCAATTTAACAATGTCTGAAAAGG R: GAATAGTTCTGGGAGACACATCG	Primer design
	TWIST	F: CTCAAGAGGTCGTGCCAATC R: CCCAGTATTTTTATTTCTAAAGGTGTT	Primer design

**Table 5:** Antibodies used in Westernblot analysis

Antibody	Dilution	Company
RXFP1	1:1000	Abnova
RXFP4	1:1000	Sigma-Aldrich
CD31	1:1000	Abcam
TGFβ1	1:1000	Abcam
Smad2/3	1:1000	Cell Signaling

## Abbreviations

AAC: ascending aortic constriction
α-SMA: smooth muscle α-actin
ATII: angiotensin 2
CD31: cluster of differentiation 31 (platelet endothelial cell adhesion molecule)
cDNA: complementary deoxyribonucleic acid
ChIP: chromatin immunoprecipitation
Col1α(I): alpha-1 type I collagen
DAPI: 4′,6-diamidino-2-phenylindole
DAPT: N-[N-(3,5-Difluorophenacetyl)-L-alanyl]-S-phenylglycine t-butyl ester
DNA: deoxyribonucleic acid
DSP: dual-specificity phosphatase
ECG: echocardiography
EF: ejection fraction
EndMT: endothelial to mesenchymal transition
eNOS: endothelial nitric oxide synthase
ERBB3: human epidermal growth factor receptor 3
FS: fractional shortening
FZD7: frizzled-7
GAPDH: glyceraldehyde 3-phosphate dehydrogenase
GSK3B: glycogen synthase kinase 3 beta
HCAEC: human coronary artery endothelial cell
HES1: hairy and enhancer of split-1
Jag1: jagged 1
LVIDd: diastolic left ventricular inner diameter
LVIDs: systolic left ventricular inner diameter
MCEC: mouse cardiac endothelial cell
MEM: minimal essential medium
MMP: matrix metalloprotease
NF-κB: nuclear factor kappa-light-chain-enhancer of activated B cells
NICD: notch protein intracellular domain
No.: Numero sign
NP40: nonyl phenoxypolyethoxylethanol
n.s.: no significance
PBS: phosphate-buffered saline
PWTd: diastolic posterior wall thickness
PWTs: systolic posterior wall thickness
qPCR: quantitative polymerase chain reaction
RAC1: ras-related C3 botulinum toxin substrate 1
RLN: relaxin
RXFP: relaxin family peptide receptor
SEM: standard error of mean
SDS-PAGE: sodium dodecyl sulfate-polyacrylamide gel electrophoresis
Ser: Serelaxin
shRNA: short hairpin ribonucleic acid
siRNA: small interfering ribonucleic acid
TAC: transverse aortic constriction
TBST: tris-buffered saline and tween
TGF: transforming growth factor
Vol d: diastolic left ventricular volume
Vol s: systolic left ventricular volume
WGA: wheat germ agglutinin
