# Supplementary material for: Serelaxin alleviates cardiac fibrosis through inhibiting endothelial-to-mesenchymal transition via RXFP1
Source: Theranostics. 2020 Mar 4;10(9):3905–24. doi: 10.7150/thno.38640 (PMC7086357; doi:10.7150/thno.38640)
Supplement: Supplementary file 1 — Supplementary figures. [file thnov10p3905s1.pdf]

Serelaxin alleviates cardiac fibrosis through inhibiting endothelial-to-mesenchymal transition via RXFP1

Tim Wilhelmi[1,3‡], Xingbo Xu, PhD[1,3‡], Xiaoying Tan, PhD[2,3], Melanie S. Hulshoff, MSc[1,3,4], Sabine Maamari, MSc[1], Samuel Sossalla, MD[1,3,5], Michael Zeisberg, MD[2,3] and Elisabeth M. Zeisberg, MD[1,3*]

-Supplementary Information-

[1] Department of Cardiology and Pneumology, University Medical Center of Göttingen, Georg-August University, Göttingen, Germany

[2] Department of Nephrology and Rheumatology, University Medical Center of Göttingen, Georg-August University, Göttingen, Germany

[3] DZHK (German Centre for Cardiovascular Research, partner site Göttingen, Germany)

[4] Laboratory for Cardiovascular Regenerative Medicine, Department of Pathology and Medical Biology, University of Groningen, Groningen, The Netherlands

[5] Department of Internal Medicine II, University Medical Center Regensburg, Regensburg, Germany

‡Authors contributed equally to this work

*Corresponding author

Correspondence to: Dr. Elisabeth Zeisberg, M.D.
Department of Cardiology and Pneumology
University Medical Center of Göttingen,
Georg-August-University
Robert-Koch-Str. 40
37075 Göttingen, Germany
Telephone: +49-(0)551-3920076
Email: elisabeth.zeisberg@med.uni-goettingen.de

A

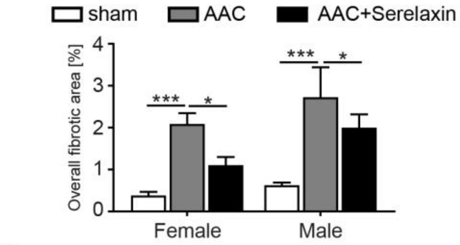

	sham		AAC+vehicle		AAC+Serelaxin	
	Female	Male	Female	Male	Female	Male
day 0	3	3	14	14	15	14
day 28	3	3	8	7	12	11
Survival (%)	100	100	57.14286	50	80	78.57142
Significance	n.s.		n.s. (p=0.8208)		n.s. (p=0.7994)	

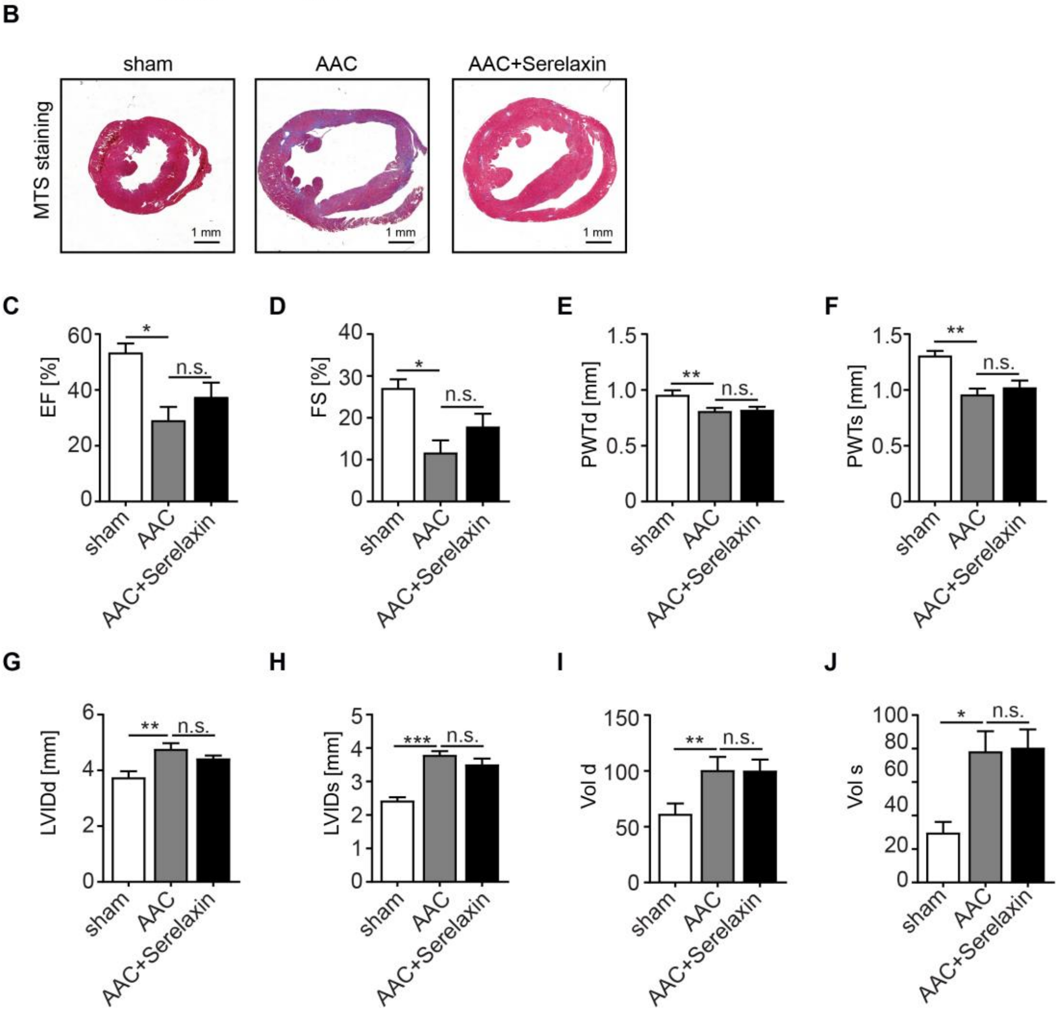

Figure S1. AAC-operated mouse hearts showed an enlarged phenotype. (A) Bar graphs showing the differences of total fibrotic area between male and female groups (left panel). Table summarizing the difference between male and female groups (right panel). (B) Masson's trichrome stained images of sham, AAC-operated and AAC-operated + Serelaxin-treated mouse hearts. The AAC-operated heart shows an enlarged phenotype and an increased area of fibrosis as compared to sham. Serelaxin reduced the area of fibrosis but did not reverse the enlarged phenotype. (C) – (J) Bar graphs show the echocardiographic parameters at 28 days after AAC operation. EF, FS, PWTd and PWTs were significantly reduced upon AAC surgery and were not significantly reversed by administration of Serelaxin. LVIDd, LVIDs, Vol d and Vol s were significantly increased upon AAC surgery but were not significantly reduced by application of Serelaxin. EF: ejection fraction; FS: fractional shortening; PWTd: diastolic posterior wall thickness, PWTs: systolic posterior wall thickness; LVIDd: diastolic left ventricular inner diameter; LVIDs: systolic left ventricular inner diameter; Vol d: diastolic left ventricular volume; Vol s: systolic left ventricular volume. Student t-test was used for single comparison and one-way ANOVA with Bonferroni post-hoc analysis was used for multiple group comparisons. Gene expression and associated error bars, representing mean ±SEM, n≥3, n.s. no significance, * $p<0.05$, ** $p<0.01$, *** $p<0.001$.

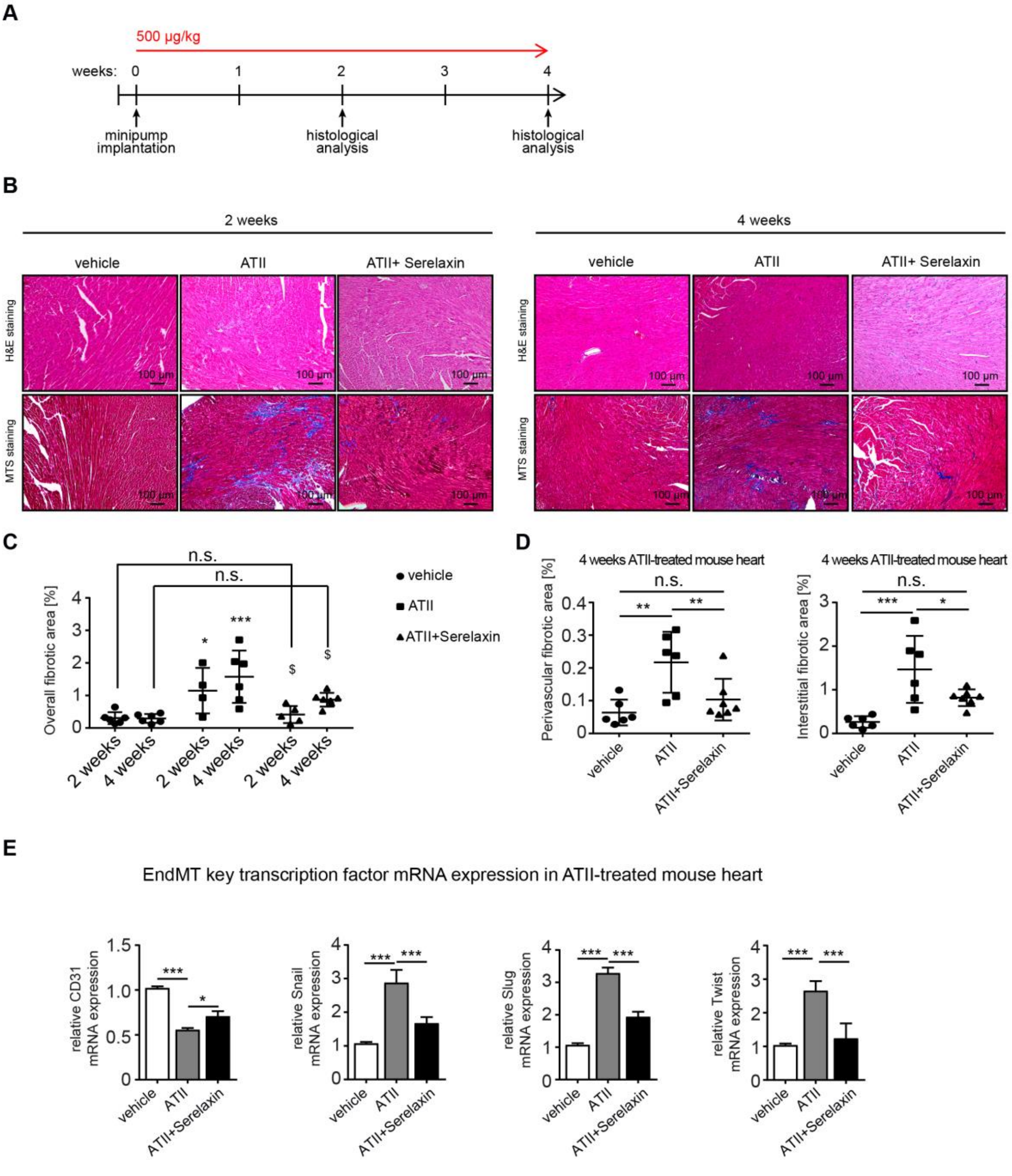

A
500 µg/kg
weeks: 0 1 2 3 4
minipump implantation
histological analysis
histological analysis
B
2 weeks
4 weeks
vehicle
ATII
ATII+ Serelaxin
H&E staining
MTS staining
100 µm
C
n.s.
n.s.
Overall fibrotic area [%]
vehicle
ATII
ATII+Serelaxin
2 weeks
4 weeks
D
4 weeks ATII-treated mouse heart
Perivascular fibrotic area [%]
Interstitial fibrotic area [%]
vehicle
ATII
ATII+Serelaxin
E
EndMT key transcription factor mRNA expression in ATII-treated mouse heart
relative CD31 mRNA expression
relative Snail mRNA expression
relative Slug mRNA expression
relative Twist mRNA expression

Figure S2. Serelaxin ameliorates Angiotensin II (ATII) - induced cardiac fibrosis. (A) Experimental plan for ATII-induced cardiac fibrosis. (B) HE and Masson's Trichrome Staining microphotography of vehicle, ATII and ATII + Serelaxin-treated mouse hearts showing the significant reduction of overall fibrosis in Serelaxin-treated (500 µg/kg/day) hearts compared to ATII-treated hearts at two different time points (2 weeks and 4 weeks). (C) Bar graphs showing the percentage of overall fibrotic area in the control (vehicle), ATII-treated and ATII + Serelaxin-treated mouse hearts after 2 weeks and 4 weeks. (D) Graphs showing the percentage of interstitial and perivascular fibrotic area in vehicle, ATII and ATII + Serelaxin-treated mouse hearts after 4 weeks. There is a significant reduction of interstitial and perivascular fibrosis in ATII and Serelaxin-treated (500 µg/kg/day) hearts compared to ATII-treated hearts. (E) qPCR analysis showing the relative mRNA expression level of CD31 and EndMT key regulators Snail, Slug, and Twist in vehicle, ATII and ATII + Serelaxin-treated mouse hearts. ATII-treated hearts showed an increased expression of Snail, Slug and Twist but a decreased expression of CD31 compared to vehicle-treated hearts, while ATII in combination with Serelaxin treatment reduced Snail, Slug and Twist expression and restored CD31 expression when compared to ATII-treated hearts. Student t-test was used for single comparison and one-way ANOVA with Bonferroni post-hoc analysis was used for multiple group comparisons. Gene expression and associated error bars, representing mean ±SEM, n≥3, n.s. no significance, * $p<0.05$, ** $p<0.01$, *** $p<0.001$.

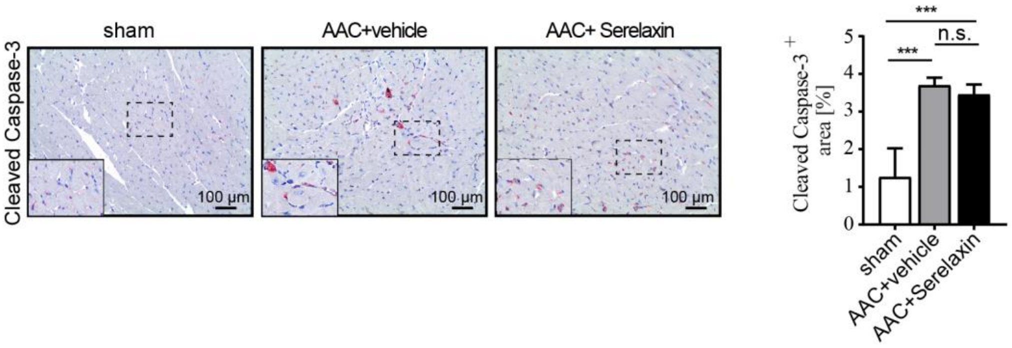

Figure S3. Serelaxin does not affect apoptosis in AAC-operated mouse hearts. Immunohistochemistry staining of cleaved Caspase 3, which is a critical executioner of apoptosis. The staining showed an increased level of cleaved Caspase 3 in AAC-operated hearts compared to sham operated hearts. Serelaxin could not significantly affect cleaved Caspase 3 levels. Student t-test was used for single comparison and one-way ANOVA with Bonferroni post-hoc analysis was used for multiple group comparisons. Error bars represent mean ±SEM, n≥3, n.s. no significance, *** $p<0.001$.

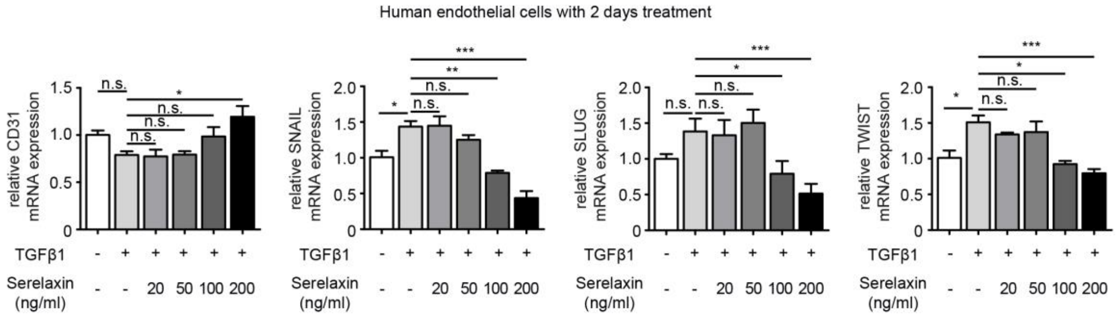

Figure S4. Serelaxin alleviates EndMT in HCAECs upon TGFβ1 treatment after 2 days. qPCR analysis showing the expression of endothelial cells marker CD31 and expression of EndMT key regulators SNAIL, SLUG, and TWIST in TGFβ1-treated HCAECs supplemented with different doses of Serelaxin after 2 days. Cells without any treatment were used as control. Serelaxin treatment significantly rescues expression of CD31 (200 ng/ml) and decreases expression of SNAIL, SLUG and TWIST (100 and 200 ng/ml). Student t-test was used for single comparison and one-way ANOVA with Bonferroni post-hoc analysis was used for multiple group comparisons. Error bars represent mean ±SEM, n≥3, n.s. no significance, * $p<0.05$, ** $p<0.01$, *** $p<0.001$.

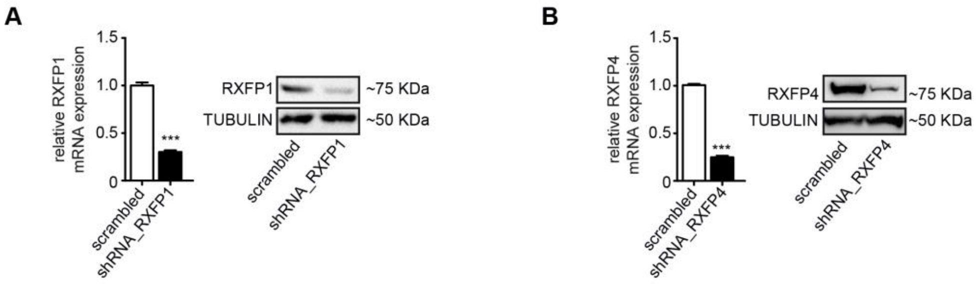

Figure S5. Validation of specifity of RXFP1- and RXFP4 shRNA knockdown. (A-B) qPCR analysis showing the relative mRNA level and Western blot analysis showing the protein level of RXFP1 (A) and RXFP4 (B) in scrambled compared to shRXFP1 and shRXFP4-mediated knockdown in HCAECs. Student t-test was used for single comparison and one-way ANOVA with Bonferroni post-hoc analysis was used for multiple group comparisons. Gene expression and associated error bars, representing mean ±SEM, n≥3, *** p<0.001.

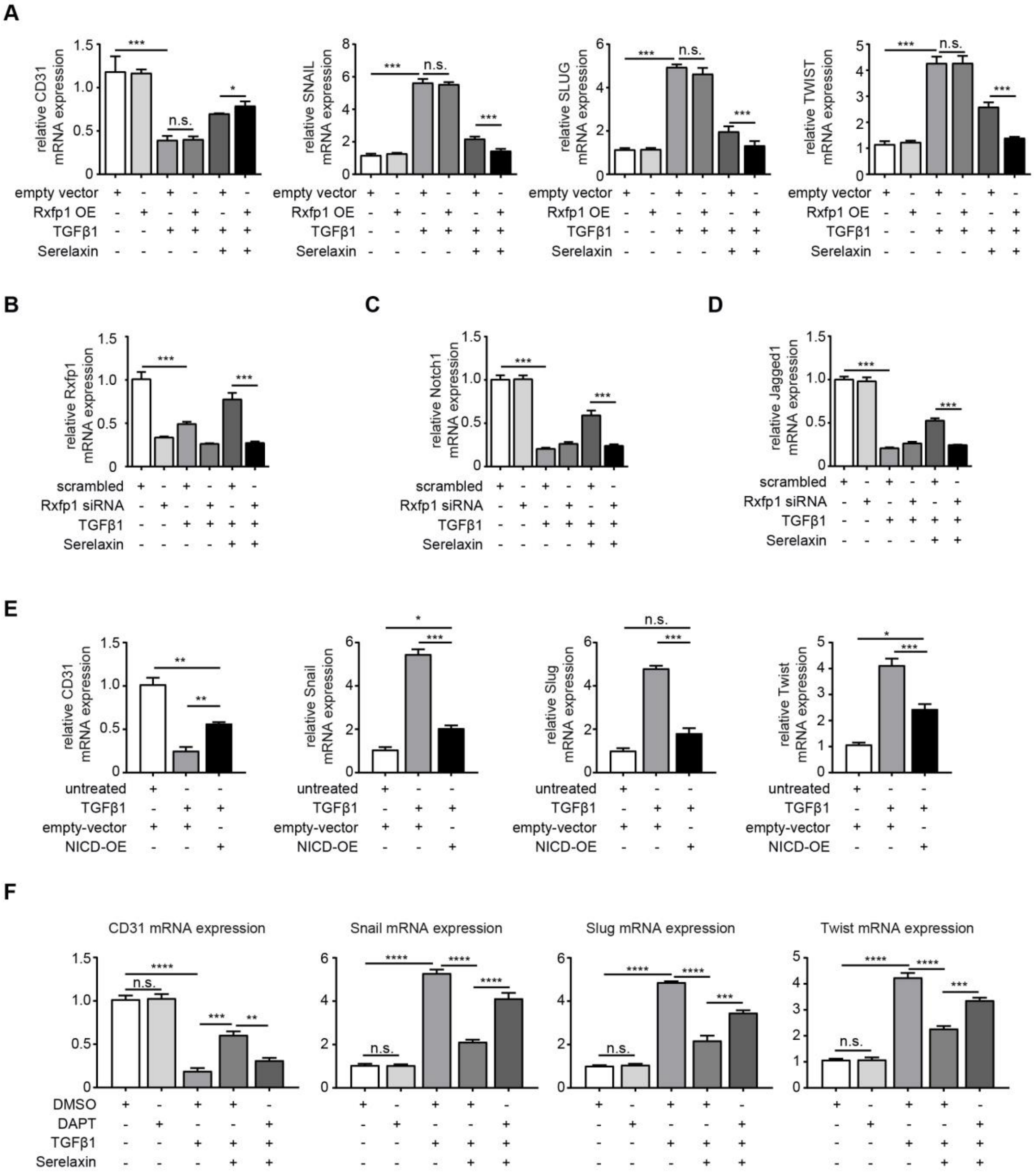
A
relative CD31 mRNA expression
relative SNAIL mRNA expression
relative SLUG mRNA expression
relative TWIST mRNA expression
empty vector
Rxfp1 OE
TGFβ1
Serelaxin
n.s.
B
C
D
relative Rxfp1 mRNA expression
relative Notch1 mRNA expression
relative Jagged1 mRNA expression
scrambled
Rxfp1 siRNA
E
untreated
empty-vector
NICD-OE
relative Snail mRNA expression
relative Slug mRNA expression
relative Twist mRNA expression
F
CD31 mRNA expression
Snail mRNA expression
Slug mRNA expression
Twist mRNA expression
DMSO
DAPT

Figure S6. Serelaxin mediates effects on EndMT by activation of the Notch1 pathway via Rxfp1. (A) qPCR analysis showing the expression of endothelial cell marker CD31 and EndMT transcriptional factors Snail, Slug and Twist in TGFβ1 and Serelaxin-treated MCECs upon Rxfp1 overexpression. Cells transfected with empty vector were used as control. In cells with Rxfp1 overexpression, Serelaxin enhanced its inhibitory effect on TGFβ1-induced EndMT as shown by increased CD31 expression and decreased expression of EndMT key transcriptional factor genes Snail, Slug and Twist. (B-D) qPCR analysis showing the expression of Rxfp1, the Notch1 receptor and its ligand Jagged1 in TGFβ1- and Serelaxin-treated, shRNA knocked down MCECs. Scrambled cells were used as control. The expression of all three genes was partly restored in Serelaxin-supplemented, TGFβ1-treated cells, but not in Rxfp1 knockdown cells. (E) qPCR analysis showing that NICD overexpression could partially block TGFβ1-induced EndMT as shown by increased expression of endothelial marker gene CD31 and decreased expression of EndMT transcriptional factors Snail, Slug and Twist. Cells transfected with an empty vector were used as control. (F) qPCR analysis showing an increased expression of endothelial marker gene CD31 and decreased expression of EndMT transcriptional factors Snail, Slug and Twist upon treatment with TGFβ1 and Serelaxin compared to TGFβ1 alone. When the cells were additionally treated with Notch-inhibitor DAPT this effect was abolished. Cells transfected with DMSO were used as control. Student t-test was used for single comparison and one-way ANOVA with Bonferroni post-hoc analysis was used for multiple group comparisons. Gene expression and associated error bars, representing mean ±SEM, $n \geq 3$, n.s. no significance, * $p<0.05$, ** $p<0.01$, *** $p<0.001$, **** $p<0,0001$.

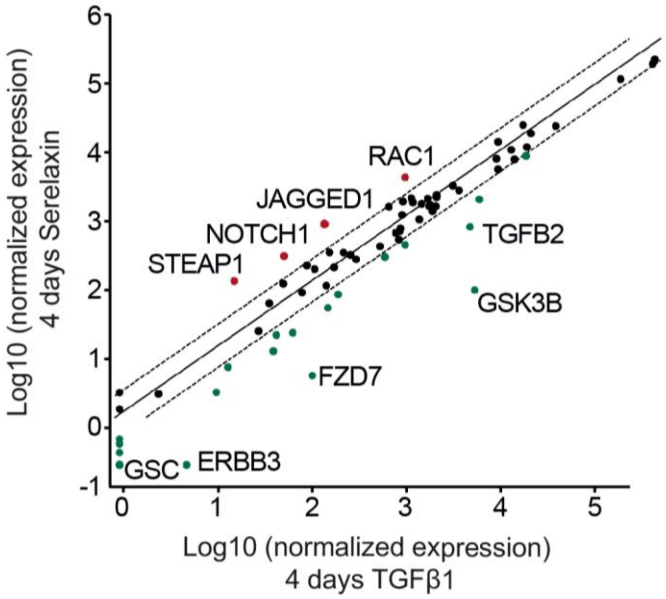

Figure S7. Identification of Serelaxin-regulated genes in TGFβ1-treated HCAECs. (A) Scatter plot showing the genes with altered mRNA expression level in only TGFβ1-treated (x-axis) or only Serelaxin-treated (y-axis) HCAECs. STEAP1, NOTCH1, JAGGED1, RAC1, DSP, GSC, ERBB3, FZD7, GSK3B and TGFB2 are significantly regulated are significantly regulated by Serelaxin treatment, which is shown by separation from dot lines (cut-off by 4 folds).
